# Targeting fibrotic signaling pathways by EGCG as a therapeutic strategy for uterine fibroids

**DOI:** 10.1038/s41598-023-35212-6

**Published:** 2023-05-25

**Authors:** Md Soriful Islam, Maclaine Parish, Joshua T. Brennan, Briana L. Winer, James H. Segars

**Affiliations:** Division of Reproductive Sciences and Women’s Health Research, Department of Gynecology and Obstetrics, Johns Hopkins Medicine, 720 Rutland Ave, Ross Building, Room 624, Baltimore, MD 21205 USA

**Keywords:** Drug discovery, Drug discovery and development

## Abstract

Fibrosis is characterized by excessive accumulation of extracellular matrix, which is a key feature of uterine fibroids. Our prior research supports the tenet that inhibition of fibrotic processes may restrict fibroid growth. Epigallocatechin gallate (EGCG), a green tea compound with powerful antioxidant properties, is an investigational drug for uterine fibroids. An early phase clinical trial showed that EGCG was effective in reducing fibroid size and its associated symptoms; however, its mechanism of action(s) has not been completely elucidated. Here, we probed effects of EGCG on key signaling pathways involved in fibroid cell fibrosis. Viability of myometrial and fibroid cells was not greatly affected by EGCG treatment (1–200 µM). Cyclin D1, a protein involved in cell cycle progression, was increased in fibroid cells and was significantly reduced by EGCG. EGCG treatment significantly reduced mRNA or protein levels of key fibrotic proteins, including fibronectin (*FN1*), collagen (*COL1A1*), plasminogen activator inhibitor-1 (*PAI-1*), connective tissue growth factor (*CTGF*), and actin alpha 2, smooth muscle (*ACTA2*) in fibroid cells, suggesting antifibrotic effects. EGCG treatment altered the activation of YAP, β-catenin, JNK and AKT, but not Smad 2/3 signaling pathways involved in mediating fibrotic process. Finally, we conducted a comparative study to evaluate the ability of EGCG to regulate fibrosis with synthetic inhibitors. We observed that EGCG displayed greater efficacy than ICG-001 (β-catenin), SP600125 (JNK) and MK-2206 (AKT) inhibitors, and its effects were equivalent to verteporfin (YAP) or SB525334 (Smad) for regulating expression of key fibrotic mediators. These data indicate that EGCG exhibits anti-fibrotic effects in fibroid cells. These results provide insight into mechanisms behind the observed clinical efficacy of EGCG against uterine fibroids.

## Introduction

Uterine fibroids (also known as leiomyomas) are the most common benign tumors of the uterus. Fibroids are highly prevalent in African American women compared to Caucasians. Fibroids are present in approximately 80% of black women and nearly 70% of white women by age of 50^1,2^. Although the majority of fibroids are asymptomatic, nearly 25% women experience significant clinical symptoms. Associated symptoms include heavy and abnormal uterine bleeding, pelvic pain or pressure, infertility or reproductive dysfunction^2,3^. Medical treatments have been approved for treatment of fibroids; however, many treatments are partially effective or are associated with side effects^4^. For example, ulipristal acetate is effective in reducing fibroid size and associated symptoms^5^. However, concerns about the risk of rare but serious liver injury with ulipristal acetate treatment have been raised^6^. Recently, elagolix^7^ and relugolix^8^ have been shown to be effective to reduce heavy menstrual bleeding in women with uterine fibroids. These treatments are associated with hypoestrogenic effects (especially decreases in bone mineral density). While these may be mitigated by using addback therapy^7,8^, they are not approved for long-term use exceeding 2 years. Surgery is the alternative options for women with symptomatic fibroids. But loss of fertility and surgery- associated adverse effects have a negative impact on women’s quality of life. Moreover, the annual cost associated with fibroid management is significant as it is estimated to be between $5.9 billion and $34.4 billion in the United States alone^9^.

Fibroids are composed of an increased mass of extracellular matrix (ECM), characteristic of their fibrotic nature^10^. This excessive accumulation of ECM, may be triggered, at least in part, by an inflammatory response, tissue injury, and angiogenesis^11^. Growth factors and cytokines play an important role in promoting pathological fibrosis^11,12^. It has been shown that TGF-β members (such as TGF-β and activin A) can increase the production of ECM proteins in uterine fibroids^13–15^, suggesting their critical role in fibrosis. PAI-1 (plasminogen activator inhibitor 1) and CTGF (connective tissue growth factor) are known to be downstream targets of TGF-β/activin A and can participate in mediating fibrosis^16^. The α-SMA (actin alpha 2, smooth muscle) is a functional marker of myofibroblasts, and is also induced by TGF-β1^17^. Myofibroblasts play a critical role in regulating tissue homeostasis. However, an inappropriate function of myofibroblasts (such as failure to undergo apoptosis) can cause pathological fibrosis^18^. While the role of Smad signaling in fibroid cells is well documented^14,19^, our recent data suggest that Hippo/YAP pathway is altered in fibroids and is involved in inducing fibrotic phenotype in fibroid cells^20^. The fibrotic role of other signaling pathways such as β-catenin, JNK (c-Jun N-terminal Kinase), and AKT/PKB (protein kinase B) in fibroid cells has not been fully elucidated^21,22^.

Epigallocatechin gallate (EGCG) is a type of catechin isolated from green tea. EGCG has been reported to induce antiproliferative and apoptotic effects in uterine leiomyoma cells^23–25^. In addition, the therapeutic effects of EGCG have been demonstrated in animal models. Female athymic nude mice were given 1.25 mg EGCG/day (in drinking water) for 4 and 8 weeks^25^. Results showed that EGCG treatment significantly reduced the volume and weight of tumors^25^. EGCG treatment (200 or 400 mg of EGCG/kg of diet) also significantly reduced the size and number of leiomyomas of the oviduct in Japanese quail after 12 months^26^. In a randomized controlled pilot clinical trial, women with symptomatic fibroids were given green tea extract (45% EGCG) (800 mg/day) for 4 months^27^. Results showed that EGCG reduced fibroid volume by 32.6% and reduced specific symptom severity by 32.4%, compared to the placebo group^27^. Notably, adverse events such as endometrial hyperplasia were not observed in the EGCG treated group^27^.

In a recent randomized phase I study, the hepatic safety profile of EGCG in healthy reproductive-aged women with and without fibroids was studied^28^. Patients were given 720 mg of EGCG alone or in combination with clomiphene citrate or letrozole for 5 days and investigators found no signs of drug induced liver injury^28^. The promising effects observed have led to clinical trials to examine the safety and efficacy of EGCG (NCT04177693; NCT05409872; NCT0536008). These observations suggest that EGCG might be a clinically safe and effective natural compound for fibroid treatment. Although EGCG appears effective, the underlying mechanisms of action on uterine fibroids have not been fully elucidated. Here, we report that EGCG induced antifibrotic effects in uterine fibroid cells. EGCG altered multiple signaling pathways involved in mediating fibrosis in uterine fibroid cells, suggesting the possibility of combination treatments for fibroid therapy.

## Results

### Effect of EGCG on cell viability and cyclin D1 expression in myometrial and uterine fibroid cells

**Figure 1:**
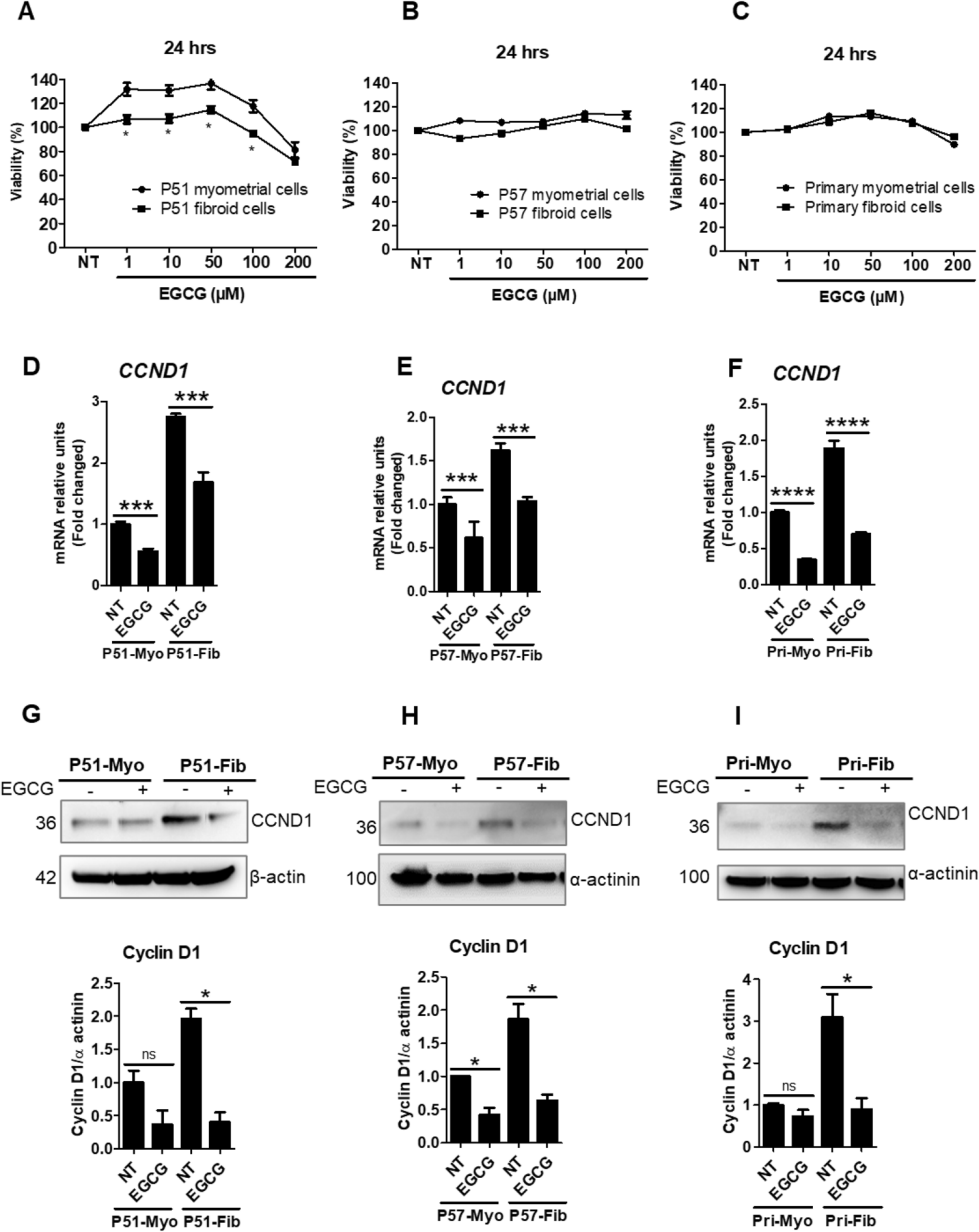
EGCG treatment differentially affected viability of myometrial and fibroid cells and reduced expression of proteins involved in cell cycle progression. (**A**–**C**) Cell viability tests were performed using an MTS assay after EGCG treatment at concentrations from 1 to 200 µM for 24 h in P51 myometrial and fibroid cells (**A**), P57 myometrial and fibroid cells (**B**), and primary myometrial and fibroid cells (**C**). Untreated control cells (without EGCG) are indicated in the MTS assay (1**A**–**C**) by “NT,” indicating no treatment. (**D**–**F**) Effects of EGCG on a regulator of cell cycle progression, Cyclin D1 (*CCND1*) transcript levels, in P51 myometrial and fibroid cells (**D**), P57 myometrial and fibroid cells (**E**), and primary myometrial and fibroid cells (**F**). (**G**–**I**) Western blotting analysis of cyclin D1 expression in P51 myometrial and fibroid cells (**G**), P57 myometrial and fibroid cells (**H**), and primary myometrial and fibroid cells (**I**) treated with EGCG (100 μM) for 24 h. NT = untreated control cells. Full immunoblots related to Fig. 1 are shown in Figs. S1–S3. Lower panels (**G**–**I**) show protein quantification. Membranes were cut into several pieces (based on the molecular weight of proteins of interest) prior to hybridization with primary antibodies during blotting. Data are presented as mean ± SEM of two to four independent experiments. *p < 0.05, **p < 0.01, ***p < 0.001, ****p < 0.0001.

An MTS assay was used to assess the effects of EGCG on cell viability of myometrial and uterine fibroid cells. Three matched myometrial and fibroid cell types were used for this study. These included P51 myometrial and fibroid cells, P57 myometrial and fibroid cells, and primary cultures of myometrial and fibroid cells. Cells were treated with EGCG at various concentrations (1, 10, 50, 100 and 200 µM) for 24 h. The percentage absorbance curves show the differential effect of EGCG on cell viability of P51 myometrial and fibroid cells. We found that viability of P51 fibroid cells was reduced by 5% at 100 µM and 28% at 200 µM of EGCG, compared to control (100%), and cells were unaffected by low doses (1–50 µM) (Fig. 1A). On the other hand, normal P51 myometrial cell viability was increased at 1–100 µM and only decreased at 200 µM (19% reduction) (Fig. 1A). For P57 and primary myometrial and fibroid cells, cell viability was not greatly altered by EGCG treatment (Fig. 1B–C). Based on the viability curves and a previous report^24^, we selected the dose of EGCG (100 µM) for the next set of experiments. Cyclin D1 (*CCND1*) is a critical protein involved in the cell cycle progression. We measured both mRNA and protein levels of cyclin D1 in myometrial and fibroid cells. We found that mRNA levels of *CCND1* were increased in P51 fibroid cells by 2.8-fold (Fig. 1D), in P57 fibroid cells by 1.6-fold (Fig. 1E), and in primary fibroid cells by 1.9-fold (Fig. 1F), compared to respective patient matched myometrial cells. EGCG treatment significantly reduced mRNA levels of *CCND1* by 39% in P51 fibroid cells, by 36% in P57 fibroid cells, and by 63% in primary fibroid cells, compared to untreated control fibroid cells (100%) (Fig. 1D–F). Similar to transcript levels, protein levels of cyclin D1 were significantly reduced by EGCG treatment in all three matched myometrial and fibroid cell lines (Fig. 1G–I). The maximum reduction (80%) was observed in P51 fibroid cells (Fig. 1G), followed by 71% in primary fibroid cells (Fig. 1I), and 66% in P57 fibroid cells (Fig. 1H) following EGCG treatment, compared to control (100%). The EGCG effects on cyclin D1 were not significant in P51myometrial cells (Fig. 1G) and in primary myometrial cells (Fig. 1I), but were significant in P57 myometrial cells (Fig. 1H). Overall, the results of transcript and protein levels of cyclin D1 suggest an antiproliferative effect of EGCG in uterine fibroid cells.

### EGCG treatment reduced expression of ECM proteins in uterine fibroid cells

**Figure 2:**
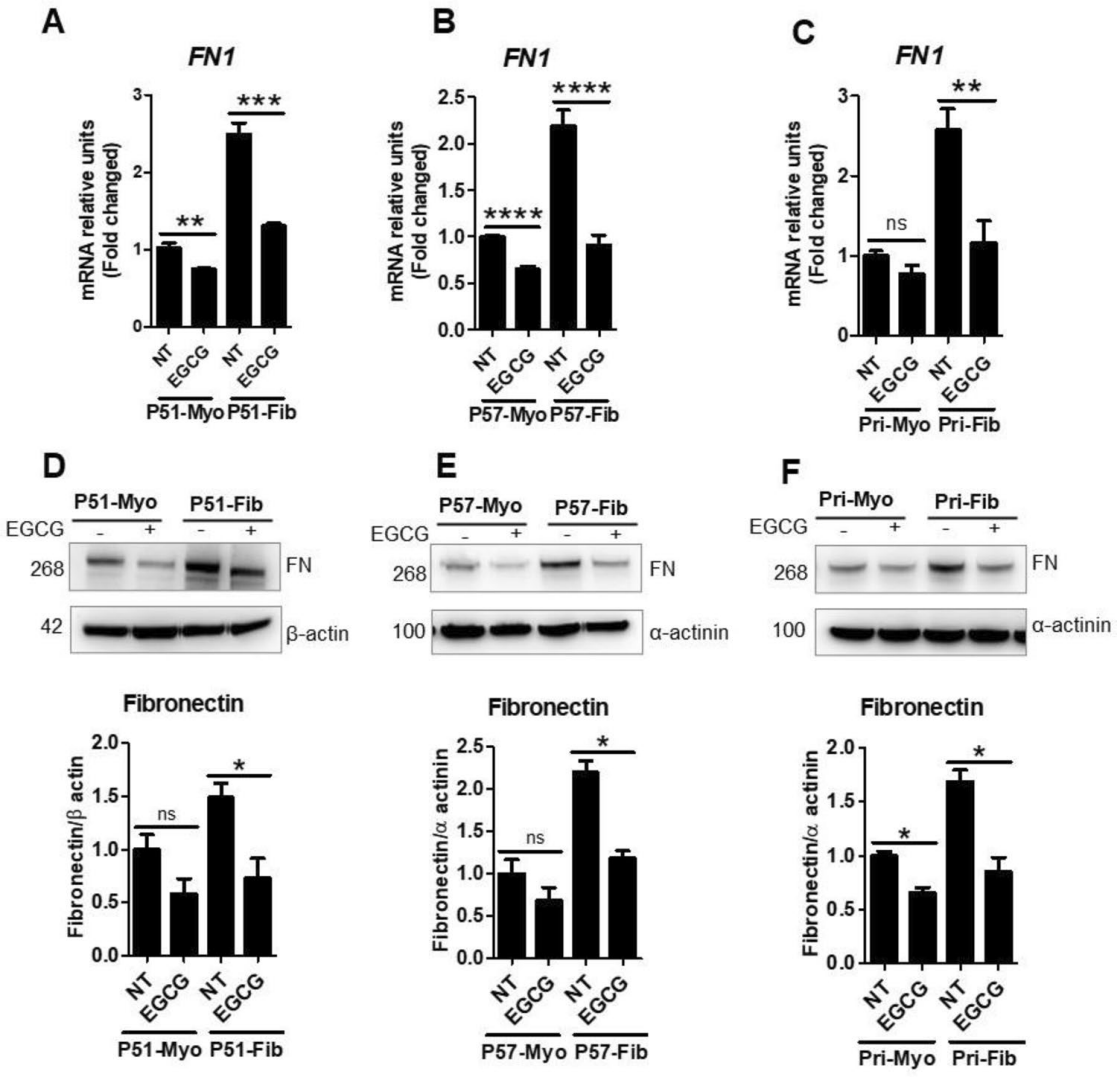
Levels of fibronectin mRNA and protein expression were reduced by EGCG treatment. (**A**–**C**) Transcript levels of fibronectin (*FN1*) in P51 myometrial and fibroid cells (**A**), P57 myometrial and fibroid cells (**B**), and primary myometrial and fibroid cells (**C**) treated with EGCG (100 μM) for 24 h. (**D**–**F**) Protein levels of fibronectin in P51 myometrial and fibroid cells (**D**), P57 myometrial and fibroid cells (**E**), and primary myometrial and fibroid cells (**F**) treated with EGCG (100 μM) for 24 h. Full immunoblots related to Fig. 2 are shown in Figs. S4–S6. Lower panels (**D**–**F**) show protein quantification. Membranes were cut into several pieces (based on the molecular weight of proteins of interest) prior to hybridization with primary antibodies during blotting. NT = untreated control cells (not exposed to EGCG). Data are presented as mean ± SEM of two to four independent experiments. *p < 0.05, **p < 0.01, ***p < 0.001, ****p < 0.0001.

Since functionally important ECM proteins are critical to fibrosis, we quantified mRNA and protein levels of such proteins, including fibronectin (*FN1*). Fibronectin is known to be overexpressed in uterine fibroids^29^. As expected, we also found that the mRNA levels of *FN1* were higher in P51 fibroid cells by 2.5-fold (Fig. 2A), in P57 fibroid cells by 2.2-fold (Fig. 2B), in primary fibroid cells by 2.6-fold (Fig. 2C), compared to myometrial cells, which were significantly reduced by EGCG treatment as by 39%, 59%, and 55%, respectively, compared to respective control (100%). While the mRNA levels of fibronectin were unaffected by EGCG treatment in primary myometrial cells (Fig. 2C), levels were reduced by EGCG treatment in P51 and P57 myometrial cells (Fig. 2A–B). Similar to mRNA levels, the protein levels of fibronectin were also higher (1.5 to 2.2-fold) in all three sets of fibroid cells, compared to respective myometrial cells (Fig. 2D–F). EGCG treatment significantly reduced fibronectin protein levels by 46–52% in fibroid cells, compared to untreated control fibroid cells (100%) (Fig. 2D–F). Fibronectin protein levels were not significantly reduced in P51 and P57 myometrial cells (Fig. 2D–E) but were significant in primary myometrial cells (Fig. 2F). Our extended experiment examined the effect of EGCG on the expression of collagens, which are major components of fibroid ECM. Real time qPCR data showed that *COL1A1* (collagen type I alpha 1 chain) mRNA levels were 1.64-fold higher in P57 fibroid cells, and 2.6-fold higher in primary fibroid cells, compared to their respective myometrial counter parts (Supplementary Fig. 1A–B). EGCG treatment reduced *COL1A1* mRNA levels by 51.8% in P57 fibroid cells, and by 59.2% in primary fibroid cells, compared to untreated control cells (Supplementary Fig. 1A–B). Overall, these results suggest that EGCG exhibited a differential effect on fibroid compared to myometrial cells and revealed antifibrotic effects as evident by inhibition of fibronectin and collagen production in fibroid cells.

### EGCG treatment reduced expression of downstream and upstream mediators of fibrosis in uterine fibroid cells

**Figure 3:**
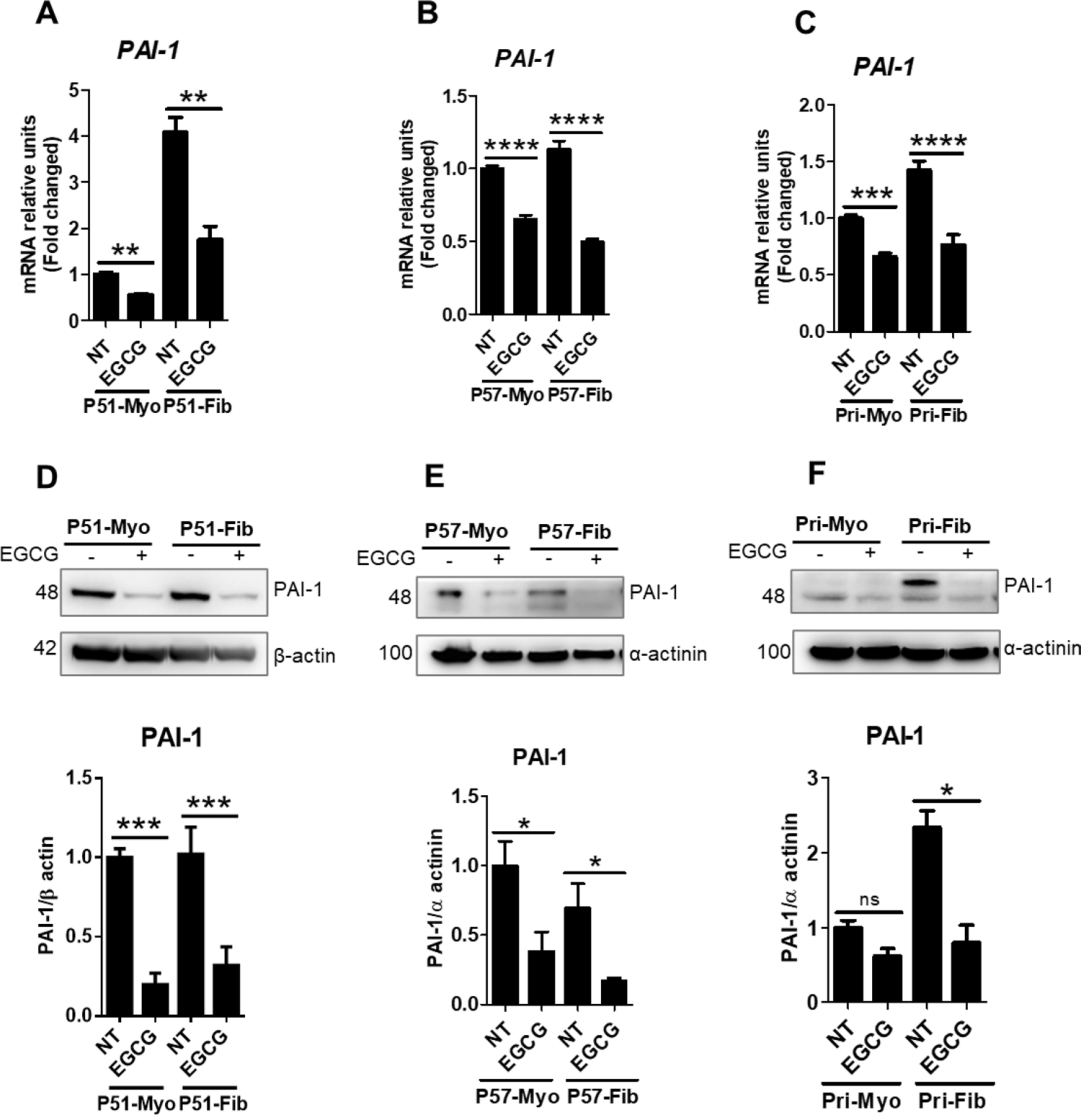
EGCG treatment reduced PAI-1 mRNA and protein expression. (**A**–**C**) Transcript levels of *PAI-1* in P51 myometrial and fibroid cells (**A**), P57 myometrial and fibroid cells (**B**), and primary myometrial and fibroid cells (**C**) were treated with EGCG (100 μM) for 24 h. (**D**–**F**) Protein levels of PAI-1 in P51 myometrial and fibroid cells (**D**), P57 myometrial and fibroid cells (**E**), and in primary myometrial and fibroid cells (**F**) treated with EGCG (100 μM) for 24 h. Protein levels were normalized by β actin and α-actinin. Full immunoblots related to Fig. 3 are shown in Figs. S7–S9. Lower panels (**D**–**F**) show protein quantification. Membranes were cut into several pieces (based on the molecular weight of proteins of interest) prior to hybridization with primary antibodies during blotting. NT = untreated control cells. Data are presented as mean ± SEM of two to four independent experiments. *p < 0.05, **p < 0.01, ***p < 0.001.

**Figure 4:**
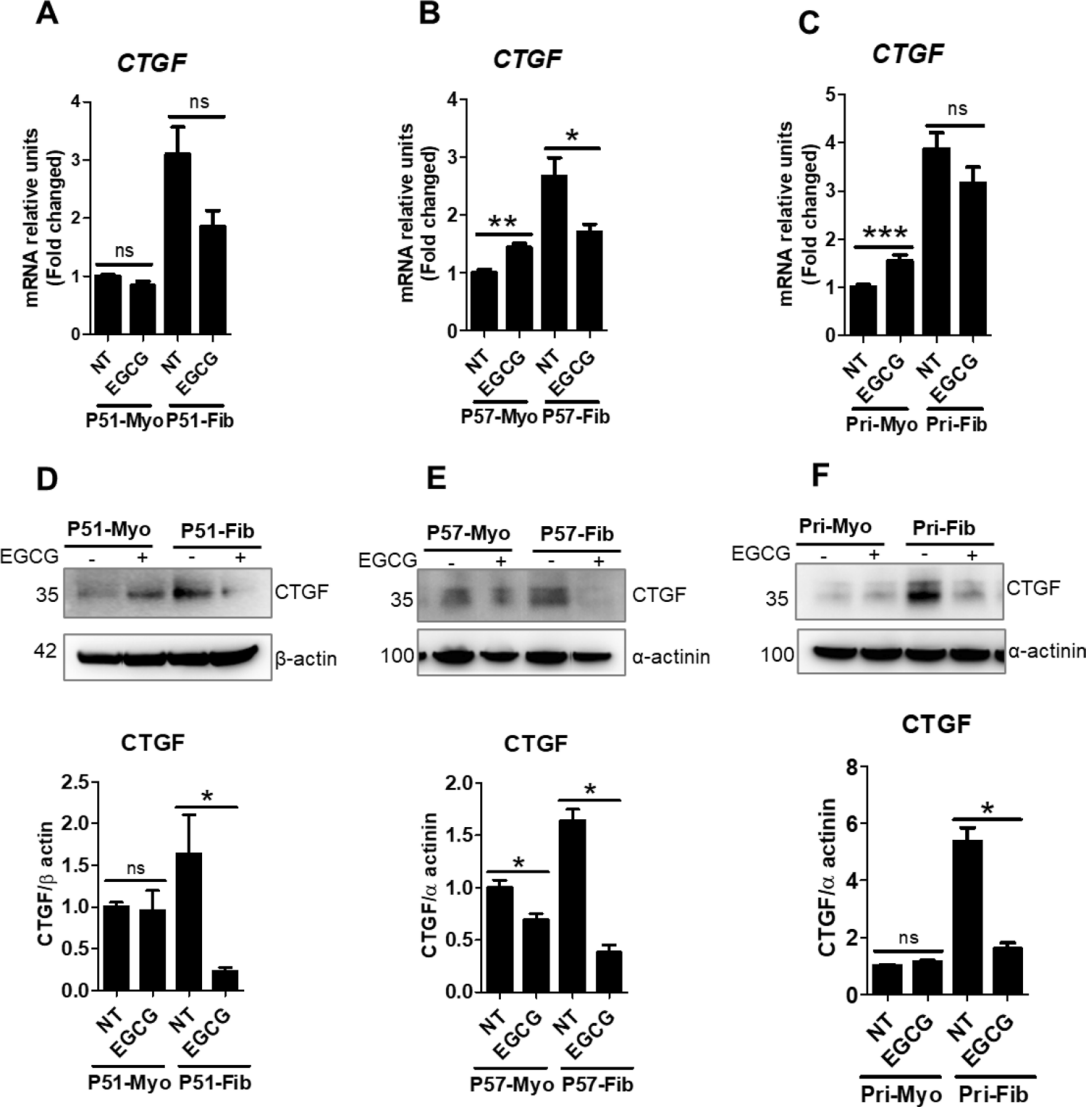
EGCG treatment reduced transcripts and protein levels for CTGF. (**A**–**C**) Transcript levels of *CTGF* in P51 myometrial and fibroid cells (**A**), P57 myometrial and fibroid cells (**B**), and primary myometrial and fibroid cells (**C**) treated with EGCG (100 μM) for 24 h. (**D**–**F**) The protein levels of CTGF in P51 myometrial and fibroid cells (**D**), P57 myometrial and fibroid cells (**E**), and primary myometrial and fibroid cells (**F**) treated with EGCG (100 μM) for 24 h. Protein levels were normalized by β actin and α-actinin. Full immunoblots related to Fig. 4 are shown in Figs. S10–S12. Lower panels (**D**–**F**) show protein quantification. Membranes were cut into several pieces (based on the molecular weight of proteins of interest) prior to hybridization with primary antibodies during blotting. NT = untreated control cells. Data are presented as mean ± SEM of two to four independent experiments. *p < 0.05, **p < 0.01, ***p < 0.001.

Next, we focused on mRNA and protein levels of PAI-1 and CTGF in response to EGCG treatment. In a previous study, we reported that PAI-1 and CTGF are overexpressed in uterine fibroid cells^20^. Here we found that the mRNA levels of *PAI-1* were 4.1-fold higher in P51 fibroid cells (Fig. 3A), 1.13-fold in P57 fibroid cells (Fig. 3B), and 1.4-fold in primary fibroid cells (Fig. 3C), compared to myometrial cells. EGCG treatment significantly reduced (46–57%) mRNA levels of *PAI-1* across all three sets of fibroid cells, compared to untreated control fibroid cells (100%) (Fig. 3A–C). Western blot analysis showed that the protein levels of PAI-1 were higher (2.3-fold) in primary fibroid cells, compared to myometrial cells (Fig. 3F) but not in immortalized P51 and P57 fibroid cells (Fig. 3D–E). We observed that EGCG greatly reduced (66–76%) protein levels of PAI-1 in all three fibroid cells, compared to untreated control fibroid cells (100%) (Fig. 3D–F). Next, we quantified the expression levels of CTGF in fibroid and myometrial cells after treatment with EGCG. We found that the basal levels of *CTGF* mRNA were higher (2.7–3.9-fold) in all three fibroid cells, compared to myometrial cells (Fig. 4A–C). EGCG treatment significantly reduced (36%) mRNA levels of *CTGF* in P57 fibroid cells (Fig. 4B), but not in P51 fibroid cells (Fig. 4A), or in primary fibroid cells (Fig. 4C), compared to untreated control fibroid cells (100%). We found higher levels of CTGF protein in P51 fibroid cells (1.63-fold) (Fig. 4D), in P57 fibroid cells (1.64-fold) (Fig. 4E), and primary fibroid cells (2.33-fold) (Fig. 4F). EGCG treatment induced a significant reduction in protein levels of CTGF in all three sets of fibroid cells (Fig. 4D–F). The maximum reduction of CTGF protein levels by EGCG was 86% in P51 fibroid cells (Fig. 4D), followed by 77% in P57 fibroid cells (Fig. 4E), and 66% in primary fibroid cells (Fig. 4F), compared to untreated control fibroid cells (100%). Notably, effects of EGCG on CTGF protein levels were not significant in P51 myometrial and primary myometrial cells but they were statistically significant in P57 myometrial cells (Fig. 4D–F).

In addition to CTGF and PAI-1, α-SMA is also considered as an important mediator in fibrosis^30^. The expression of α-SMA is considered a functional marker for myofibroblasts, which contribute to fibrosis^31,32^. As an upstream target of CTGF, PAI-1 and α-SMA^33–35^, we also included profibrotic growth factors activin A (*INHBA*) and TGF-β (*TGFB)* in our studies. An active TGF-β1 plays important role in converting fibroblasts into contractile myofibroblasts^36^. Experiments revealed that mRNA levels of α-SMA (*ACTA2*) were 3.8-fold higher in P57 fibroid cells, which was reduced by 69% with EGCG treatment, compared to control (100%) (Supplementary Fig. 2A). Western blot analysis showed that the protein levels of α-SMA were higher (4.9-fold) in P57 fibroid cells, compared to P57 myometrial cells (Supplementary Fig. 2B). EGCG treatment severely reduced (71%) α-SMA protein expression in P57 fibroid cells but not in P57 myometrial cells, compared to control (100%) (Supplementary Fig. 2B). We found higher mRNA levels of *INHBA* (4.3-fold), *TGFB1* (2.0-fold), and *TGFB2* (5.1-fold) in P51 fibroid cells, compared to normal myometrial cells (Supplementary Fig. 2C–E). EGCG treatment significantly reduced mRNA levels of *INHBA* by 60%, *TGFB1* by 40%, and *TGFB2* by 50% in P51 fibroid cells, compared to their respective untreated controls (100%) (Supplementary Fig. 2C–E). Overall, these results suggest that fibroid cells highly express several key mediators of fibrosis, which were effectively reduced by treatment with EGCG.

### EGCG treatment altered the activation of fibrotic signaling pathways in uterine fibroid cells

**Figure 5:**
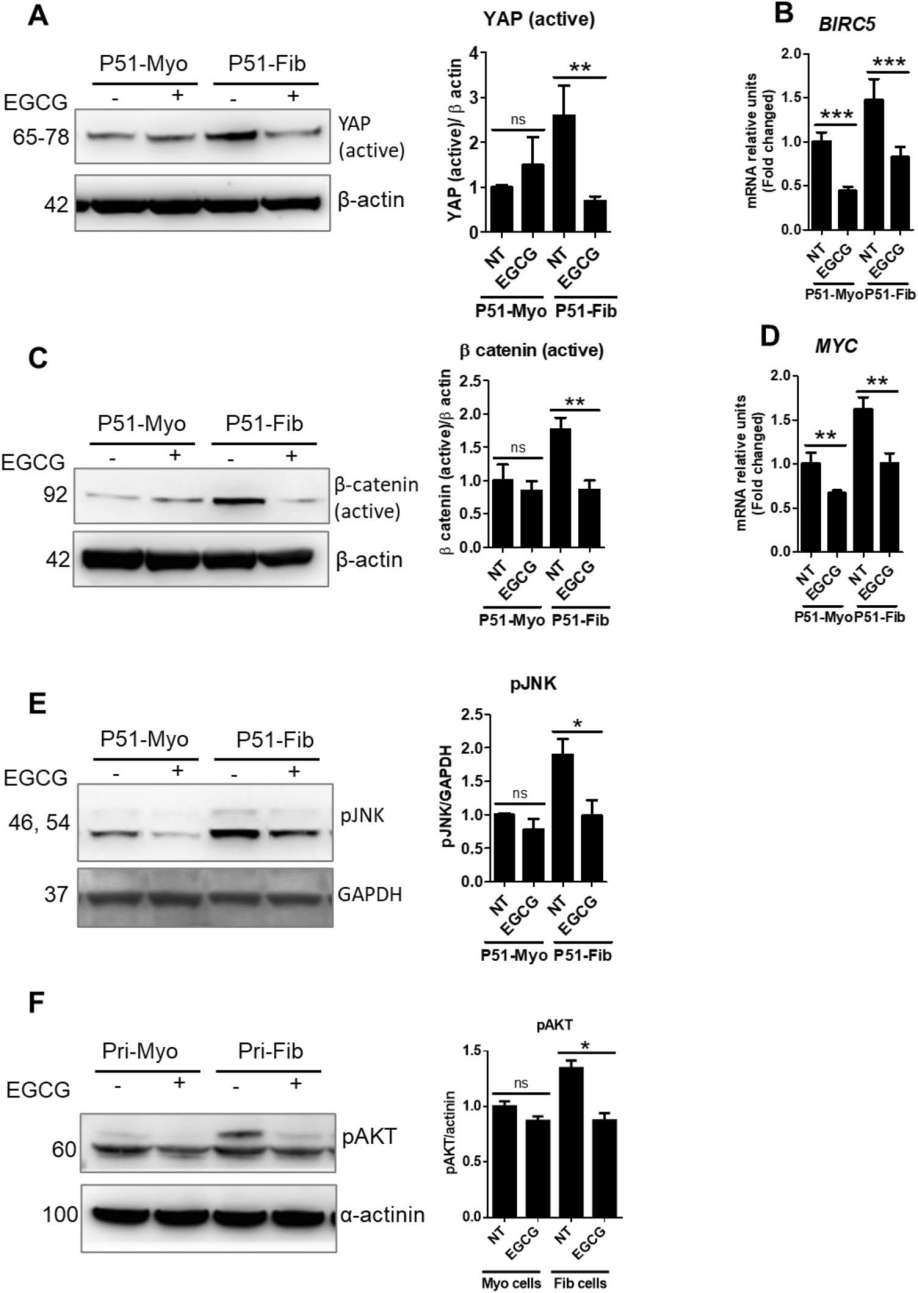
EGCG effects on fibrotic signaling pathways in fibroid cells. (**A**) The protein levels of non-phospho-YAP (active) in P51 myometrial and fibroid cells treated with EGCG (100 µM) for 24 h. (**B**) The transcript levels of *BIRC5* in P51 myometrial and P51 fibroid cells. (**C**) The protein levels of non-phospho-β-catenin (Ser45) in P51 myometrial and fibroid cells treated with EGCG (100 µM) for 24 h. (**D**) The transcript levels of *MYC* in P51 myometrial and P51 fibroid cells treated with EGCG (100 µM) for 24 h. (**E**) The protein levels of phospho-JNK (Thr183/Tyr185) in P51 myometrial and fibroid cells treated with EGCG (100 µM) for 24 h. (**F**) The protein levels of phospho-AKT (Ser473) in primary myometrial and fibroid cells treated with EGCG (100 µM) for 24 h. Protein levels were normalized by β actin, GAPDH, and α-actinin. Full immunoblots related to Fig. 5 are shown in Figs. S13–S16. Membranes were cut into several pieces (based on the molecular weight of proteins of interest) prior to hybridization with primary antibodies during blotting. NT = untreated control cells. Data are presented as mean ± SEM of two to seven independent experiments. *p < 0.05, **p < 0.01, ***p < 0.001.

To identify the direct targets of EGCG in uterine fibroid cells, we tested the effects of EGCG on different signaling pathways, including YAP, Smad, β-catenin, JNK, and AKT. The fibrotic role of YAP and Smad has been reported in different organs^16,37,38^ as well as in uterine fibroids^14,19,20,39^. However, the fibrotic roles of β-catenin, JNK, and AKT are not fully understood in uterine fibroids^16^. Our recent data suggest that Hippo signaling (YAP activation) is involved in producing ECM proteins as well as transcription of profibrotic genes in fibroid cells^20,39^. To investigate if EGCG might alter Hippo/YAP signaling, we treated fibroid and myometrial cells with EGCG at 100 µM for 24 h. We quantified proteins levels of main transcriptional effector of Hippo/YAP signaling, YAP (non-phospho YAP; transcriptionally active). Western blot analysis showed that the protein levels of non-p-YAP were 2.6-fold higher in P51 fibroid cells, compared to myometrial cells (Fig. 5A), suggesting an activation of YAP signaling in fibroid cells. EGCG selectively reduced protein levels of non-p-YAP by 73% in P51 fibroid cells, compared to untreated control fibroid cells (100%) (Fig. 5A). The protein levels of non-p-YAP were unaffected by EGCG treatment in normal myometrial cells (Fig. 5A). We also found that EGCG treatment regulated *BIRC5* expression in fibroid cells. *BIRC5* (Baculoviral IAP Repeat Containing 5, also known as survivin) is a YAP-responsive gene^40^, which is known to inhibit apoptosis and promote cell proliferation^41^. We found that the basal mRNA levels of *BIRC5* were 1.5-fold higher in P51 fibroid cells, compared to P51 myometrial cells, which was significantly reduced (45%) by EGCG treatment, compared to untreated control (100%) (Fig. 5B). Overall, these results suggests that the antifibrotic effects of EGCG is mediated, at least in part, by alteration of fibrotic signaling involving YAP signaling.

As previously reported^14,19^, Smad signaling is involved in mediating fibrotic effects in uterine fibroid cells. To investigate if EGCG could affect Smad signaling, we treated fibroid and myometrial cells with EGCG at 100 µM for 24 h. We quantified proteins levels of main transcriptional effector of Smad signaling, phospho-Smad2 (active). Western blot analysis showed that the protein levels of pSmad2 were 1.8-fold higher in P51 fibroid cells, compared to P51 myometrial cells (Supplementary Fig. 3), suggesting an activation of Smad signaling in fibroid cells. Treatment of P51 fibroid cells with EGCG showed no reduction on pSmad2 levels (Supplementary Fig. 3). In contrast, the levels of pSmad2 were significantly increased by EGCG treatment in P51 myometrial cells (Supplementary Fig. 3). Although the role of Smad signaling in fibroid cell fibrosis is well understood, EGCG was not able to alter the activation of this signaling.

While the role of β-catenin in the regulation of cell proliferation, differentiation, and apoptosis is well-known, the role of β-catenin in mediating fibrosis in uterine fibroid cells has not been clearly demonstrated^21^. To study whether EGCG could regulate β-catenin signaling, we treated P51 myometrial and fibroid cells with EGCG at 100 µM for 24 h. We quantified proteins levels of the transcriptionally active form of β-catenin (non-phospho-β-catenin). We found that protein levels of active β-catenin were higher (1.8-fold) in P51 fibroid compared to P51 myometrial cells (Fig. 5C), suggesting an activation of β-catenin signaling in fibroid cells. EGCG treatment decreased β-catenin (active) protein levels by 51% in fibroid cells, compared to untreated control fibroid cells (100%) (Fig. 5C). Notably, in P51 myometrial cells, the protein levels of β-catenin (active) were not significantly affected by EGCG treatment (Fig. 5C). We also quantified the downstream target of β-catenin signaling, *MYC* (MYC proto-oncogene, bHLH transcription factor). We found that the basal mRNA levels of *MYC* were 1.6-fold higher in P51 fibroid cells, compared to P51 myometrial cells (Fig. 5D). EGCG treatment reduced *MYC* transcript levels by 38% in fibroid cells, compared to untreated control fibroid cells (100%) (Fig. 5D). Overall, these results suggest that β-catenin signaling can be targeted by EGCG in fibroid cells.

JNK and AKT signaling pathways are commonly associated with cell growth and survival^42^. To study if EGCG could regulate JNK signaling, we treated P51 myometrial and fibroid cells with EGCG at 100 µM for 24 h. We quantified proteins levels of active form of JNK. We found that protein levels of phospho-JNK (Thr183/Tyr185) (active) were higher (1.9-fold) in P51 fibroid compared to P51 myometrial cells (Fig. 5E), suggesting an activation of JNK signaling in fibroid cells. EGCG treatment significantly reduced phospho-JNK (Thr183/Tyr185) levels by 48% in P51 fibroid cells, compared to untreated control fibroid cells (100%) (Fig. 5E). Remarkably, in P51 myometrial cells, the protein levels of phospho-JNK (Thr183/Tyr185) were not significantly affected by EGCG treatment (Fig. 5E). Overall, these results suggest that EGCG can effectively target JNK signaling.

To study if EGCG could regulate AKT signaling, we treated primary myometrial and fibroid cells with EGCG at 100 µM for 24 h. We quantified proteins levels of active form of AKT. We found that protein levels of phospho-AKT (Ser473) (active) were higher (1.3-fold) in primary fibroid cells, compared to primary myometrial cells (Fig. 5F), suggesting an activation of AKT signaling in fibroid cells. EGCG treatment significantly reduced phospho-AKT (Ser473) levels by 35% in primary fibroid cells but not in primary myometrial cells, compared untreated control (100%) (Fig. 5F). These results suggest that EGCG can target AKT signaling.

### Comparative effects of EGCG with synthetic inhibitors against fibroid cell fibrosis

**Figure 6:**
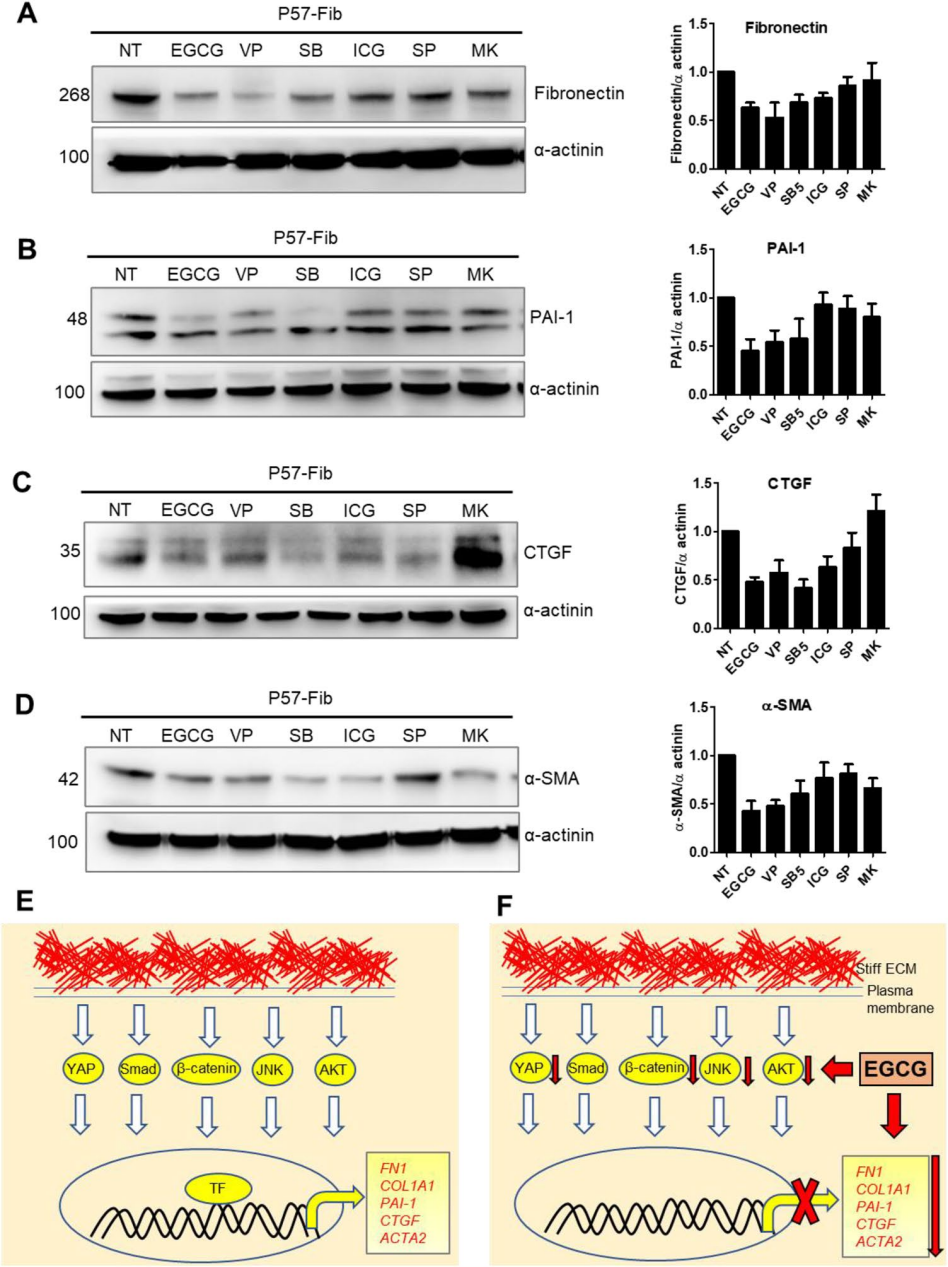
Comparative effects of EGCG with signaling pathway-specific inhibitors proteins involved in fibroid cell fibrosis. (**A**–**D**, left panel) Western blotting analysis of fibronectin (**A**), PAI-1 (**B**), and CTGF (**C**), α-SMA (**D**) in P57 fibroid cells were untreated (NT), or treated with either EGCG (100 µM); verteporfin = VP (1 µM); SB525334 = SB (1 µM); ICG-001 = ICG (2.5 µM); SP600125 = SP (5 µM); MK-2206 = MK (2.5 µM) for 24 h. (**A**–**D**, right panel) graphic quantification of protein levels corresponding to left panel. Protein levels were normalized by α-actinin. Full immunoblots related to Fig. 6 are shown in Figs. S17–S20. Membranes were cut into several pieces (based on the molecular weight of proteins of interest) prior to hybridization with primary antibodies during blotting. NT = untreated control cells. Data are presented as mean ± SEM four independent experiments. (**E**–**F**) Schematic representation of signaling pathways involved in fibroid cell fibrosis (**E**) and EGCG effects (**F**).

To study the comparative effects of EGCG with synthetic inhibitors against fibroid cell fibrosis, we treated P57 fibroid cells with EGCG (100 µM; natural compound), verteporfin (1 µM; YAP inhibitor), SB525334 (1 µM; Smad inhibitor), ICG-001 (2.5 µM; β-catenin inhibitor), SP600125 (5 µM; JNK inhibitor), MK-2206 (2.5 µM; AKT inhibitor) for 24 h. The single dose for each inhibitor was selected based on the viability test (Supplementary Fig. 4). We quantified protein levels of fibronectin, PAI-1, CTGF, and α-SMA. Western blot analysis showed that EGCG reduced fibronectin expression by 40%, while verteporfin induced a maximum 62% reduction, followed by 35% by SB525334, 28% by ICG-001, 23% by SP600125, and 26% by MK-2206 in P57 fibroid cells, compared to untreated control fibroid cells (100%) (Fig. 6A). We found that EGCG induced a large (62%) reduction in PAI-1 protein levels in P57 fibroid cells, compared to untreated control (100%). However, verteporfin induced a maximum inhibition of PAI-1 levels (79%), followed by 64% by SB525334, 38% by ICG-001, 55% by SP600125, and 33% by MK-2206 in P57 fibroid cells, compared to untreated control fibroid cells (100%) (Fig. 6B). While the protein levels CTGF were increased by MK-2206 (1.2-fold), EGCG and verteporfin induced an equal 52% reduction, followed by 55% reduction by SB525334, and 30% reduction by ICG-001 and SP600125 in P57 fibroid cells, compared to untreated control fibroid cells (100%) (Fig. 6C). While EGCG and verteporfin induced an equal 68% reduction of α-SMA protein levels, SB525334 induced 43%, followed by 20% by ICG-001, 27% by SP600125, and 21% by MK-2206 in P57 fibroid cells, compared to untreated control fibroid cells (100%) (Fig. 6D). This result suggests that EGCG is more efficacious than ICG-001, SP600125 and MK-2206, and is equally effective as verteporfin and SB525334 in reducing expression of key fibrotic mediators.

## Discussion

We report for the first time that EGCG induced antifibrotic effects in uterine fibroid cells. We also found that fibrosis was mediated by multiple signaling pathways, which can be effectively blocked by EGCG treatment (Fig. 6E–F). Previously, EGCG was reported to induce antiproliferation and apoptosis in HuLM (human uterine leiomyoma) cells^23^. The antiproliferative effects of EGCG in HuLM cells were mediated by downregulation of catechol-o-methyltransferase (COMT) activity^24^. Later, its antiproliferative and apoptotic effects were confirmed in rat leiomyoma (ELT3) cells in vitro and in a nude mice model^25^. Zhang et al. reported that consumption of EGCG (1.25 mg EGCG/day for 4 and 8 weeks) by female athymic nude mice showed a significant reduction in the volume and weight of tumors^25^. Similar findings were observed against spontaneous tumors of Japanese quail with 12-month treatment of EGCG (200 or 400 mg of EGCG/kg of diet)^26^. These results of antifibrotic effects and alteration of fibrotic signaling pathways by EGCG in uterine fibroid cells represent an important addition to the understanding of EGCG action.

Previously, the effects of EGCG were studied in only immortalized fibroid cells. Here we included two varieties of immortalized fibroid and myometrial cells^43,44^, in addition to primary fibroid and myometrial cells. Viability curves revealed that EGCG differentially affected the cell viability of P51 myometrial and fibroid cells. While P51 myometrial cells grew normally at 1–100 µM of EGCG, cell proliferation of fibroid cells was slightly reduced (5%) at 100 µM and was unaffected at 1–50 µM. The viability of P57 and primary fibroid and myometrial cells was not greatly affected by EGCG. However, the reduction in transcript and protein levels of cyclin D1 (an important regulator of cell cycle progression) by EGCG at 100 µM was evident in P51 fibroid cells. Similar results were found in P57 fibroid cells and primary fibroid cells. These results suggest that EGCG may differentially affect cell cycle progression between myometrial and uterine fibroid cells, and may represent a mechanism of the clinical efficacy of EGCG against uterine fibroids.

The presence of ECM proteins, such as fibronectin in fibroids, is critical the pathogenesis of the tumors. TGF-β members (such as TGF-β and activin A) have been reported to increase deposition of ECM proteins in uterine fibroids^13–15^, which supports their critical role in mediating fibroid cell fibrosis. An ECM-rich rigid structure is thought to contribute to abnormal bleeding in the uterus^10^. Indeed, in a previous study, we reported a decrease in fibroid-related pain in patients treated with collagenase, an antifibrotic compound that specifically degrades type I and type III collagens^45^. Therefore, our approach was to test the antifibrotic effect of EGCG in fibroid cells by analyzing expression of fibronectin and collagen as well as profibrotic growth factors (activin A and TGF-β). Fibronectin and collagen levels were elevated in fibroid cells and were downregulated by EGCG treatment. EGCG treatment also decreased the levels of activin A, TGF-β1, and TGF-β2 in fibroid cells. This result suggests the potential antifibrotic effects of EGCG against uterine fibroid cells, might be mediated by downregulation of activin A/TGF-β-mediated action. Consistent with this observation, we observed that downstream targets of activin A/TGF-β, the PAI-1 and CTGF, were significantly reduced by EGCG in fibroid cells. However, we noted that phospho-Smad2 levels were not reduced following EGCG treatment in leiomyoma cells, suggesting an interesting complexity to EGCG actions. Alpha-SMA is a marker of myofibroblasts^18^. We observed that the levels of α-SMA were higher in fibroid cells, which was severely reduced by EGCG. Overall, these findings suggest that EGCG may exert antifibrotic effects via disruption of key mediators of fibrosis.

Next, we extended experiments to identify the direct targets of EGCG in uterine fibroid cells. We tested the effects of EGCG on five signaling pathways, including YAP, Smad, β-catenin, JNK, and AKT. While YAP and Smad are well-known for their fibrotic role in different organs, including fibroids^16,37,38^. The roles of β-catenin, JNK, and AKT are less studied in this context^16^. We found that uterine fibroid cells highly expressed transcriptional effectors or active proteins of YAP, Smad, β-catenin, JNK and AKT. Treatment of fibroid cells with EGCG showed a significant reduction in protein levels of non-p-YAP (active), non-p-β-catenin (active), phospho-JNK (active), and phospho-AKT (active) but not phospho-Smad2 (active), suggesting an ability of EGCG to regulate fibrosis in fibroid cells through alteration of YAP, β-catenin, JNK and AKT signaling pathways.

Finally, we conducted a comparative study to evaluate the efficiency of EGCG along with synthetic inhibitors in regulating expression of fibrotic mediators (such as fibronectin, PAI-1, CTGF, and α-SMA). Western blots showed that EGCG was more efficacious than ICG-001, SP600125 and MK-2206, and was equally effective with verteporfin and SB525334 in modulating expression of fibrotic mediators. This observation opens the possibility of combination treatment approach. Verteporfin is an FDA approved drug, which is used to eliminate abnormal blood vessels in the eye associated with conditions such as macular degeneration. Our previous reports^20^ and current data suggest that the combination treatment of EGCG and verteporfin might be a viable option for future study. SB525334 is also promising and showed tumor specific efficacy in the Eker rat model^19^. However, the therapeutic efficacy of SB525334 has been tempered because of mitogenic and antiapoptotic effects for epithelial cells in the kidney of Eker rats^19^.

In conclusion, we found that EGCG induced antifibrotic effects and altered multiple signaling pathways involved in fibrosis in fibroid cells. These results support further investigation of EGCG as treatment for fibroid growth and fibroid-associated symptoms in clinical studies. To date, one clinical trial shows that EGCG is effective in reducing fibroid volume and fibroid associated symptoms without any adverse events group^27^. Another recent phase I clinical study reported the hepatic safety profile in women with and without fibroids. In this study, no signs of drug induced liver injury was reported in women who took 720 mg of EGCG alone or in combination with clomiphene citrate or letrozole for 5 days^28^. The limited number of clinical data suggest that EGCG is well-tolerated and is not associated with liver toxicity. Our data provide insight into the underlying mechanism(s) underlying the observed effects on fibroid growth.

## Materials and methods

### Materials

EGCG (≥ 95%) was purchased from Sigma-Aldrich. EGCG was dissolved with water (cell culture grade) at 20 mM concentration and stored at − 20 °C. Verteporfin (YAP inhibitor) (cat. no. S1786), SB525334 (Smad inhibitor) (cat. no. S1476), ICG-001 (β-catenin/CBP inhibitor) (cat. no. S2662), SP600125 (JNK inhibitor) (cat. no. S1460), and MK-2206 2HCl (AKT inhibitor) (cat. no. S1078) were purchased from Selleck Chemicals. These chemicals were dissolved with DMSO and stored at − 20 °C or − 80 °C.

### Cell culture

Immortalized human uterine fibroid (P51 and P57) and patient-matched myometrial (P51 and P57) cells were collected from Minnie Malik, PhD and William Catherino M.D., Ph.D.; Uniformed Services University of the Health Sciences, Bethesda, MD). For P51 fibroid and myometrial cells, samples were collected from African American woman (44 years-old) who underwent a hysterectomy because of symptoms of bleeding and cramping. The subject had not taken GnRH analogues, such as leuprolide acetate (Lupron), cetrorelix or elagolix or hormone therapy for 3 months prior to surgery. For P57 fibroid and myometrial cells, samples were collected from an African American woman (45 years-old) who underwent a hysterectomy for heavy menstrual bleeding. The subject had not taken GnRH analogues or had been on hormone therapy for 3 months prior to surgery. No phytoestrogen was reported. Both P51 and P57 cells were immortalized by HPV-16 (Human papillomavirus 16) as previously described^43,44^. Briefly, primary cells were infected at 40–50% confluence on first passage with retrovirus stock (pSLXN virus with geneticin selection gene was a gift from Dr Rhim, Center for Prostrate Disease Research, Bethesda, MD). To enhance infection by the retroviral vector, polybrene (5 μg/mL) was added to each flask. After incubation at 37 °C for 24 h, the cells were washed once with PBS, heated to 37 °C and cultured in fresh DMEM-F12 supplemented with 10% FBS (Invitrogen). The cells were maintained at 37 °C and 5% CO_2_ for 48 h before adding fresh media containing 100 μg/mL of geneticin (Sigma-Aldrich). The cells were grown for 4 days in selection media before fresh media was added. Both myometrial and leiomyoma cells were cultured in fresh DMEM-F12 supplemented with 10% FBS (GIBCO by Life Technologies) and 100 μg/L of normocin (InvivoGen, CA, USA) at 37 °C in 95% air-5% CO_2_. The P51 and P57 cells have been characterized in studies and demonstrated to represent suitable models of fibroid and myometrial cells^14,20,44,46–51^. For primary cell usage, the institutional review board of the Johns Hopkins University reviewed and approved the study, and informed consent was obtained from study subjects. Primary myometrial and fibroid cells were isolated from patients who underwent hysterectomy at the Johns Hopkins University Hospital. The primary culture samples used in this study were from a premenopausal African American woman (age: 51 years) who underwent surgery because of symptoms of heavy menstrual bleeding with multiple fibroids. The location of the fibroids was intramural and submucous, and largest fibroid measured approximately 5.8 cm in size. The patient had not received exogenous hormones for the previous 3 months. At the time of surgery, the myometrial and leiomyoma samples were collected in Hank’s Balanced Salt Solution (HBSS) without calcium or magnesium (Thermo Fisher Scientific, Waltham, MA). The samples were washed several times with PBS (Gibco, Life Technologies Limited, Paisley, UK) to remove excess blood. Tissues were cut into small pieces and then incubated in HBSS with collagenase type IV (Worthington, Lakewood, NJ), deoxyribonuclease (DNase, Sigma-Aldrich), antibiotic–antimycotic mixture (Thermo Fisher Scientific), and HEPES buffer solution (Thermo Fisher Scientific) at 37 °C on a shaker for 4–8 h. After having digested cell suspension, it was sequentially filtered with 100 µm and 40 µm Nylon Cell Strainer (Falcon) and centrifuged at 1500 rpm twice for 5 min. After that, the supernatant was aspirated and cells were resuspended in T75 or T25 flask with fresh DMEM-F12 supplemented with 10% FBS (GIBCO by Life Technologies) and 100 μg/L of normocin (InvivoGen, CA, USA). All methods were performed in accordance with the relevant guidelines and regulations.

### MTS assay

To evaluate the effect of EGCG or inhibitors on cell viability, CellTiter 96 Aqueous One Solution cell proliferation assay (MTS) was applied. P51, P57 and primary uterine fibroid and myometrial cells were seeded as 2000 or 4000 cells per well in 96 well plate. After 24 h, cells were treated with EGCG (1, 10, 50, 100 and 200 µM), verteporfin (0.1, 0.5, 1, 2 and 5 µM), SB525334 (0.5, 1, 2.5, 5 and 10 µM), ICG-001 (0.5, 1, 5, 10 and 20 µM), SP600125 (1, 5, 10, 20 and 40 µM), and MK-2206 (0.5, 1, 2.5, 5 and 10 µM) for 24 h. MTS solution (Promega #G3588) (20 µL) was directly added to each culture well, and incubated for 2–3 h at 37 °C with 5% CO_2,_ and then recorded the absorbance at 490 nm^20^. The results of inhibitors are presented in Supplementary Fig. 4. Based on the viability analysis, we selected a single dose for further experiments.

### Quantitative real time PCR

P51, P57 and primary human uterine fibroid and myometrial cells were seeded onto 60 mm dish and cultured for 24–48 h with complete media supplemented with serum and normocin. Cells with ⁓ 70% confluency were serum starved (used serum free media) for 24 h. Serum starved P51, P57 and primary human uterine fibroid and myometrial cells were treated with EGCG at 100 µM for 24 h, and same set of cells were left untreated (control). Cells were lysed with RLT buffer subsequently extracted and purified with an RNeasy Mini Kit (Qiagen, Gaithersburg, MD, # 74,106). The concentration and purity of RNA were measured by using the NanoVue Plus (Biochrom US, Holliston, MA). The RNA was converted to cDNA (50–100 ng/µL) using iScript™ cDNA Synthesis Kit (Bio-Rad, Hercules, CA, USA #1,708,891) in a Bio-Rad Thermocycler machine. The real time qPCR was performed on LightCycler^®^ 96 System (Roche Diagnostics, Mannheim, Germany) in 96-well plate with 2.8 ng/µL of cDNA in a final volume of 10 μL, containing 1X FastStart Essential DNA green Master (Roche Diagnostics), with appropriate primer sets (Supplementary Table 1). Forward and reverse primer sequences (IDT, Coralville, Iowa) were used for determining the messenger RNA (mRNA) expression of selected genes, including, *CCND1*, *FN1*, *COL1A1*, *PAI-1*, *CTGF*, *INHBA*, *TGFB1*, *TGFB2*, *BIRC5*, *ACTA2*, *MYC*, and ribosomal protein lateral stalk subunit P0 (*RPLP0*) (Supplementary Table 1). RPLP0 was amplified under the same conditions for normalizing quantitative data. The relative mRNA expression was calculated using the ΔΔCT method and is presented as fold increase or decrease relative to control.

### Western blot

P51, P57, and primary human uterine fibroid and myometrial cells were seeded onto 100 mm dish and cultured to reach ⁓ 70% confluency, and then serum starved for 24 h. Next day, cells were treated with EGCG at 100 µM for 24 h, and cell were left untreated (control). Cells were washed with PBS and lysed with RIPA buffer (Sigma #R0278) containing protease and phosphatase inhibitor cocktail (Thermo Fisher Scientific). Protein concentrations were quantified using Pierce™ BCA Protein Assay Kit (Thermo Fisher Scientific). An equal volume (30–50 μg) of protein lysates were loaded onto 4–12% NuPAGE gels (Thermo Fisher Scientific), resolved by SDS-PAGE under reducing conditions, and then transferred to 0.2-μm nitrocellulose membranes in an X-cell II apparatus (Thermo Fisher Scientific). Ponceau S solution (Sigma #P7170) was used for the detection of protein on nitrocellulose membranes. Based on the molecular weight of proteins of interest, nitrocellulose membranes were cut prior to hybridization into several pieces. After blocking membranes with 5% non-fat-milk with TBST (1X TBS, 0.1% Tween 20) for 1 h, membranes were incubated overnight at 4 °C with primary antibodies (Supplementary Table 2). Next day, membranes were washed three times (5 min each) with TBST and then incubated with appropriate horseradish Peroxidase (HRP)-conjugated secondary antibodies as 1: 30,000 dilutions (GE Healthcare #NA934V or NA931V) with 5% non-fat-milk with TBST for ⁓ 2 h at room temperature. Membranes were washed three times (5 min each) with TBST and immunoreactive proteins were visualized using a SuperSignal™ West Pico PLUS Chemiluminescent Substrate (Thermo Fisher Scientific) in an Azure Imager c300 system (Azure Biosystems, Dublin, California). The band intensity was quantified using Java-based image processing program, Image J 1.52a and normalized against corresponding anti-β-actin or α-actinin. Since our focus was on proteins involved in fibrosis, and levels of cytoskeletal proteins can vary in response to mechanical stimulation, rather than rely solely on β-actin, we also normalized expression to α-actinin. Data are presented as fold increase or decrease relative to control.

### Statistical analysis

Cell viability data are represented as percentage mean ± SEM of 2–3 independent experiments. The fold changed data of mRNA and protein are presented as mean ± SEM of 2–7 independent experiments. The conversion of fold change to percentage was performed using a simple proportion as we did previously^52^. The baseline "fold" expression for control samples was set to “100%”. As an example, for controls, it is set at 1*100% = 100%; and if the relative expression of treatment was 0.2, then the percent expression would be 0.2*100% = 20%. Therefore, the percent change in gene or protein expression with respect to control is equal to the treatment minus the control value. Therefore, 20–100% = − 80%. The negative sign denotes a decrease in expression. The Mann–Whitney U test was used to evaluate the differences between treatment and control group. Differences were considered significant at *p* < 0.05.

## Supplementary Information

Supplementary Information 1.

Supplementary Information 2.

## Data Availability

All data associated with this study are present in the paper or the Supplementary Materials and are available from the corresponding authors on reasonable request.
