# Supplementary material for: Targeting fibrotic signaling pathways by EGCG as a therapeutic strategy for uterine fibroids
Source: Sci Rep. 2023 May 25;13:8492. doi: 10.1038/s41598-023-35212-6 (PMC10212992; doi:10.1038/s41598-023-35212-6)
Supplement: Supplementary file 1 — Supplementary Information 1. [file 41598_2023_35212_MOESM1_ESM.docx]

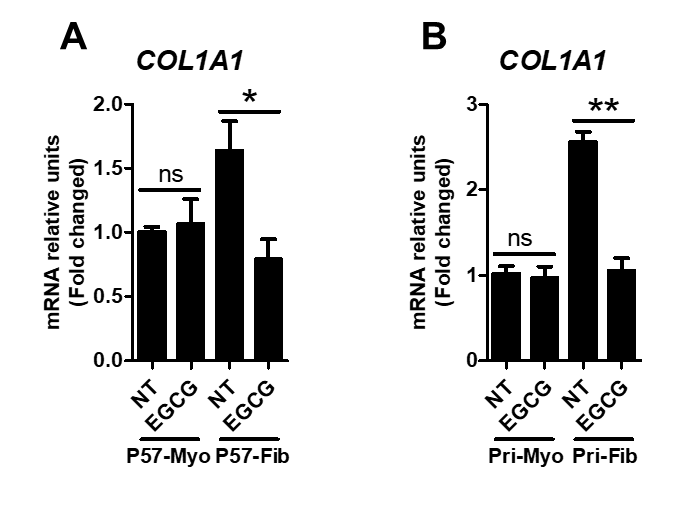


**Supplementary Figure 1.** Levels of *COL1A1* mRNA in myometrial and fibroid cells. Cells were treated with EGCG at 100 µM for 24 hrs or left untreated. Levels of COL1A1 mRNA/transcript were quantified by real time qPCR. Transcript levels of COL1A1 in P57 myometrial and fibroid cells (A), and in primary myometrial and fibroid cells (B). NT=untreated control cells (not exposed to EGCG). Data are presented as mean ± SEM of two independent experiments. *p < 0.05, **p < 0.01.


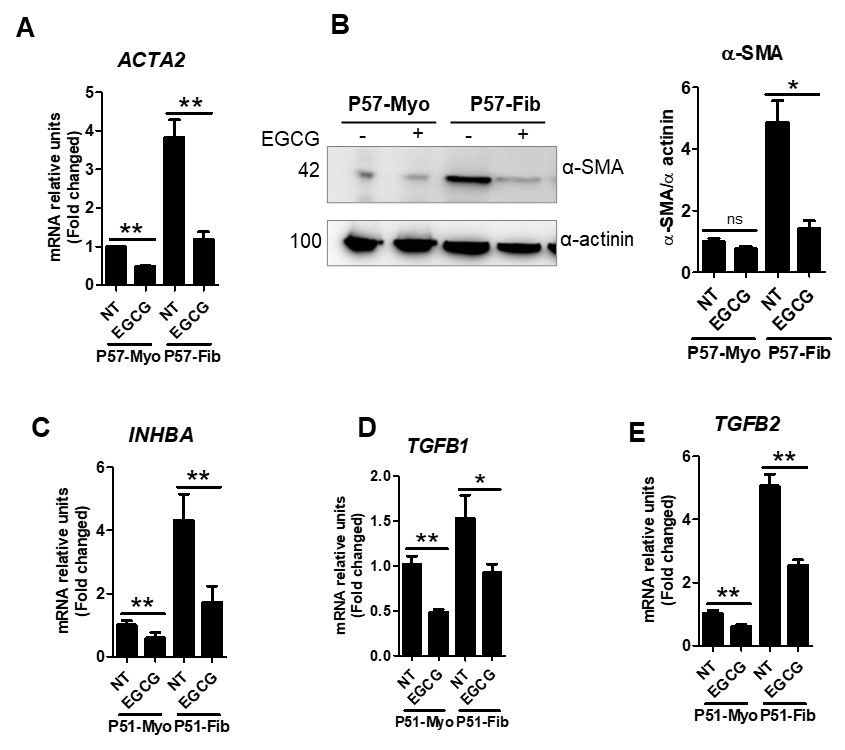


**Supplementary Figure 2.** EGCG effects on transcript levels of α-SMA and profibrotic growth factors expression in myometrial and fibroid cells. P57 or P51 myometrial and fibroid cells were treated with EGCG (100 µM) for 24 hrs. A) The transcript levels of ACTA2 in P57 myometrial and fibroid cells. B) The protein levels of α-SMA in P57 myometrial and fibroid cells. Protein levels were normalized by α-actinin. Full immunoblots related to Supplementary Figure 1 are shown in Figure S21. Membranes were cut into several pieces (based on the molecular weight of proteins of interest) prior to hybridization with primary antibodies during blotting. NT=untreated control cells. C-E) The transcript levels of *INHBA*, *TGFB1*, and *TGFB2* in P51 myometrial and fibroid cells. Data are presented as mean ± SEM of two to four independent experiments. **p <* 0.05, ***p <* 0.01.


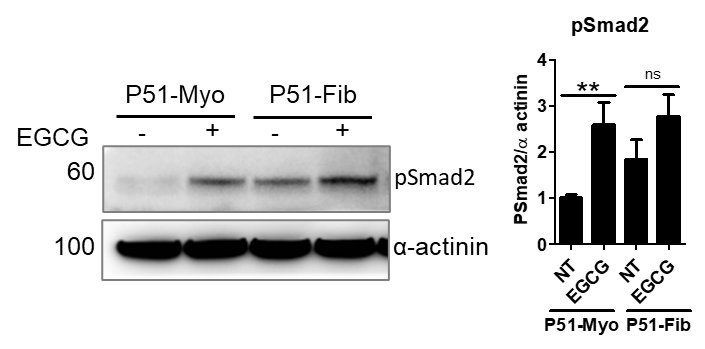


**Supplementary Figure 3.** EGCG effects on Smad signaling in fibroid cells. The protein levels of phospho Smad2 (Ser465/467) in P51 myometrial and fibroid cells treated with EGCG (100 µM) for 24 hrs. Protein levels were normalized by α-actinin. Full immunoblots related to Supplementary Figure 2 are shown in Figure S22. Membranes were cut into several pieces (based on the molecular weight of proteins of interest) prior to hybridization with primary antibodies during blotting. NT=untreated control cells. Data are presented as mean ± SEM of two to four independent experiments. *p < 0.05, **p < 0.01, ***p < 0.001.


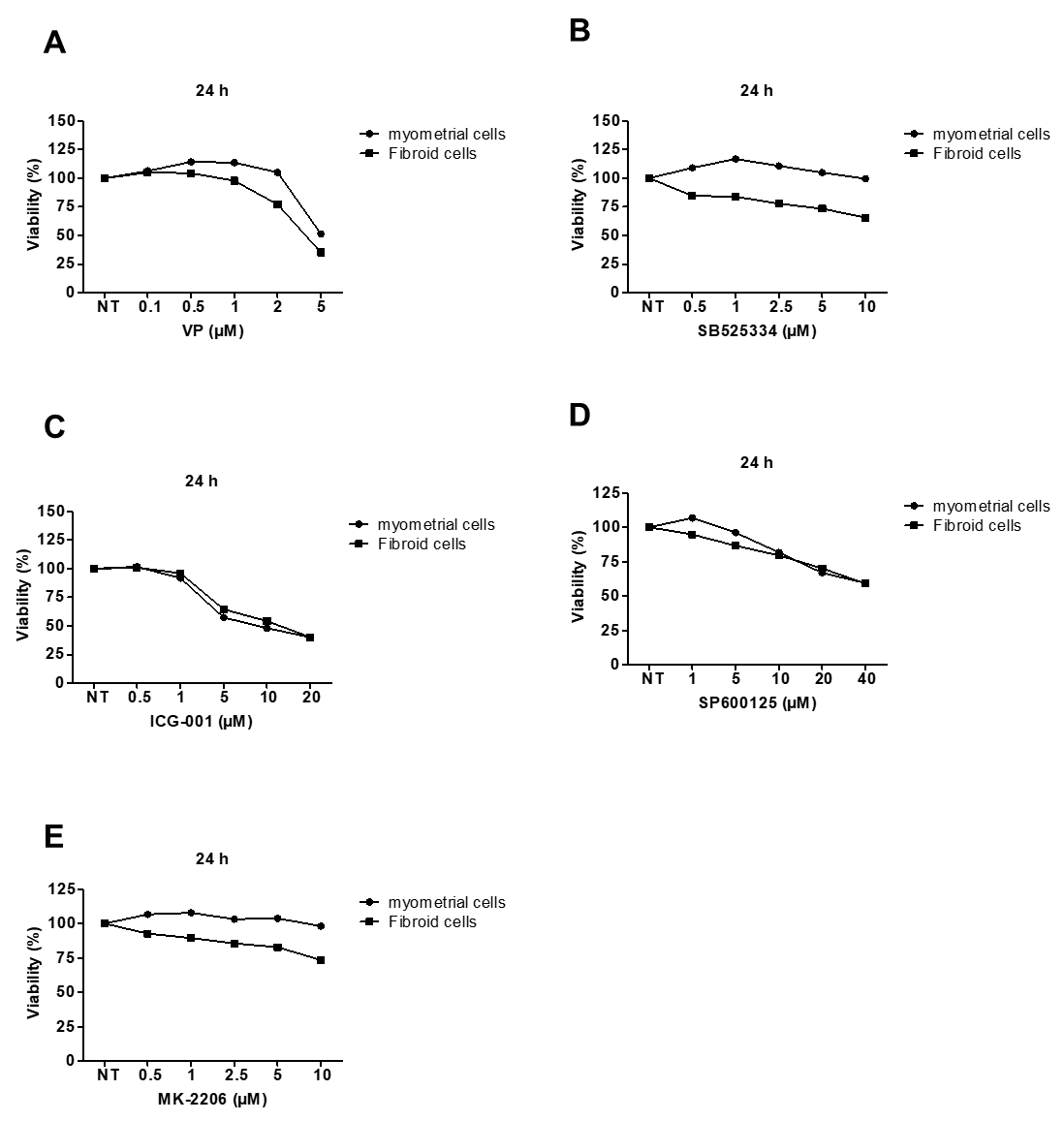


**Supplementary Figure 4**. Viability analysis of myometrial and fibroid cells of pharmacologic inhibitors used in the study. A-E) P51 myometrial and P51 fibroid cells were treated with verteporfin (0.1, 0.5, 1, 2 and 5 µM), SB525334 (0.5, 1, 2.5, 5 and 10 µM), ICG-001 (0.5, 1, 5, 10 and 20 µM), SP600125 (1, 5, 10, 20 and 40 µM), and MK-2206 (0.5, 1, 2.5, 5 and 10 µM) for 24 hrs. Cell viability test was performed using MTS assay. NT=untreated control cells. Data are presented as mean ± SEM of two independent experiments.

**Supplementary Table 1.** List of primers.

| **Gene name** | **RefSeq** | **Product length** | **Direction** | **Sequence (5'->3')** | **Length** | **Tm** | **GC%** |
| --- | --- | --- | --- | --- | --- | --- | --- |
| *ACTA2* | NM_001613.4 | 80 | Forward | TCAATGTCCCAGCCATGTAT | 20 | 56.57 | 45.00 |
|  |  |  | Reverse | CAGCACGATGCCAGTTGT | 18 | 58.34 | 55.56 |
| *BIRC5* | NM_001012271.2 | 115 | Forward | CCACTGAGAACGAGCCAGACTT | 22 | 62.24 | 54.55 |
|  |  |  | Reverse | GTATTACAGGCGTAAGCCACCG | 22 | 61.37 | 54.55 |
| *CCND1* | NM_053056.2 | 135 | Forward | GCTGCGAAGTGGAAACCATC | 20 | 59.83 | 55.00 |
|  |  |  | Reverse | CCTCCTTCTGCACACATTTGAA | 22 | 59.11 | 45.45 |
| *COL1A1* | XM_054315083.1 | 178 | Forward | GAGAGCATGACCGATGGATT | 20 | 57.46 | 50.00 |
|  |  |  | Reverse | CCTTCTTGAGGTTGCCAGTC | 20 | 58.47 | 55.00 |
| *CTGF* | NM_001901.3 | 146 | Forward | CAAGGGCCTCTTCTGTGACT | 20 | 59.31 | 55.00 |
|  |  |  | Reverse | ACGTGCACTGGTACTTGCAG | 20 | 60.88 | 55.00 |
| *FN1* | NM_212482.3 | 142 | Forward | CCATAAAGGGCAACCAAGAG | 20 | 56.38 | 50.00 |
|  |  |  | Reverse | AAACCAATTCTTGGAGCAGG | 20 | 56.20 | 45.00 |
| *INHBA* | NM_002192.4 | 156 | Forward | CATCACCTTTGCCGAGTCAG | 20 | 58.92 | 55.00 |
|  |  |  | Reverse | AGACGGATGGTGACTTTGGT | 20 | 58.94 | 50.00 |
| *MYC* | NM_002467.6 | 128 | Forward | CCTGGTGCTCCATGAGGAGAC | 21 | 62.18 | 61.90 |
|  |  |  | Reverse | CAGACTCTGACCTTTTGCCAGG | 22 | 61.13 | 54.55 |
| *PAI-1* | NM_000602.4 | 127 | Forward | GAGAACCTGGGAATGACCGAC | 21 | 60.41 | 57.14 |
|  |  |  | Reverse | TGCCACTCTCGTTCACCTCG | 20 | 62.14 | 60.00 |
| *RPLP0* | NM_001002.4 | 265 | Forward | GCGACCTGGAAGTCCAACT | 19 | 59.63 | 57.89 |
|  |  |  | Reverse | GGTCCTCCTTGGTGAACAC | 19 | 57.68 | 57.89 |
| *TGFB1* | NM_000660.7 | 124 | Forward | GGCGATACCTCAGCAACCG | 19 | 60.88 | 63.16 |
|  |  |  | Reverse | CTAAGGCGAAAGCCCTCAAT | 20 | 57.96 | 50.00 |
| *TGFB2* | NM_001135599.4 | 350 | Forward | CTGTCTACCTGCAGCACACT | 20 | 59.68 | 55.00 |
|  |  |  | Reverse | TGGGACTGTCTGGAGCACAA | 20 | 61.05 | 55.00 |

**Supplementary Table 2.** List of primary antibodies.

| **Primary antibodies** | **Cat no.** | **Company** | **Size (kDa)** | **Host/Isotype** | **WB dil.** |
| --- | --- | --- | --- | --- | --- |
| Cyclin D1 | 2922 | Cell Signaling Technology | 36 | Rabbit | 1: 1000 |
| Fibronectin | ab6584 | Abcam | 238-268 | Rabbit | 1: 10000 |
| PAI-1 | 11907 | Cell Signaling Technology | 48 | Rabbit IgG | 1: 1000 |
| CTGF | 86641 | Cell Signaling Technology | 35 | Rabbit IgG | 1: 500 |
| Non-phospho-YAP (active) | 29495 | Cell Signaling Technology | 65-78 | Rabbit IgG | 1: 500 |
| Non-p-β-catenin (active) (Ser45) | 19807 | Cell Signaling Technology | 92 | Rabbit IgG | 1: 1000 |
| Phospho-Smad2 (Ser465/467) | 3108 | Cell Signaling Technology | 60 | Rabbit IgG** | 1: 1000 |
| Phospho-SAPK/JNK (Thr183/Tyr185) | 9251 | Cell Signaling Technology | 46, 54 | Rabbit | 1: 1000 |
| Phospho-Akt (Ser473) (587F11) | 4051 | Cell Signaling Technology | 60 | Mouse IgG2b | 1: 1000 |
| α-Actinin (D6F6) | 6487 | Cell Signaling Technology | 100 | Rabbit IgG | 1: 2000 |
| α-Smooth Muscle Actin (D4K9N) | 19245 | Cell Signaling Technology | 42 | Rabbit IgG | 1: 1000 |
| β-Actin | A3854 | Sigma Aldrich | 42 | Mouse IgG | 1:30000 |
