# Supplementary material for: Targeting fibrotic signaling pathways by EGCG as a therapeutic strategy for uterine fibroids
Source: Sci Rep. 2023 May 25;13:8492. doi: 10.1038/s41598-023-35212-6 (PMC10212992; doi:10.1038/s41598-023-35212-6)
Supplement: Supplementary file 2 — Supplementary Information 2. [file 41598_2023_35212_MOESM2_ESM.docx]

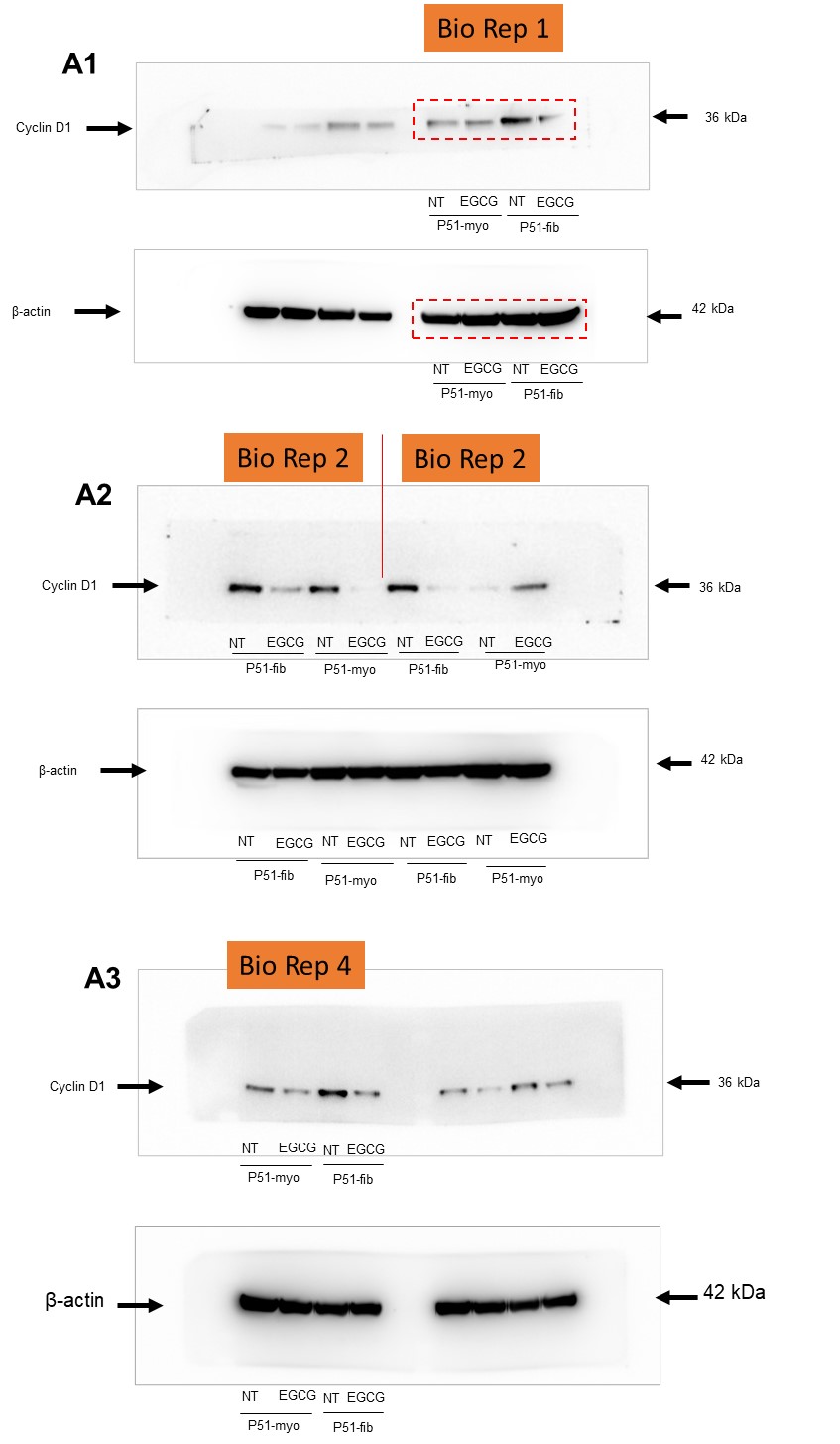


Figure S1. Full immunoblots related to Figure 1. Cyclin D1 and β-actin in P51 myometrial and fibroid cells (A1-A3). Membranes were cut into several pieces (based on the molecular weight of proteins of interest) prior to hybridization with primary antibodies during blotting. NT=untreated control cells. Biological replicates (Bio Rep) of immunoblots are presented here used for analysis. MW (in kDa) are indicated. β-actin was used as loading control to normalize the data.


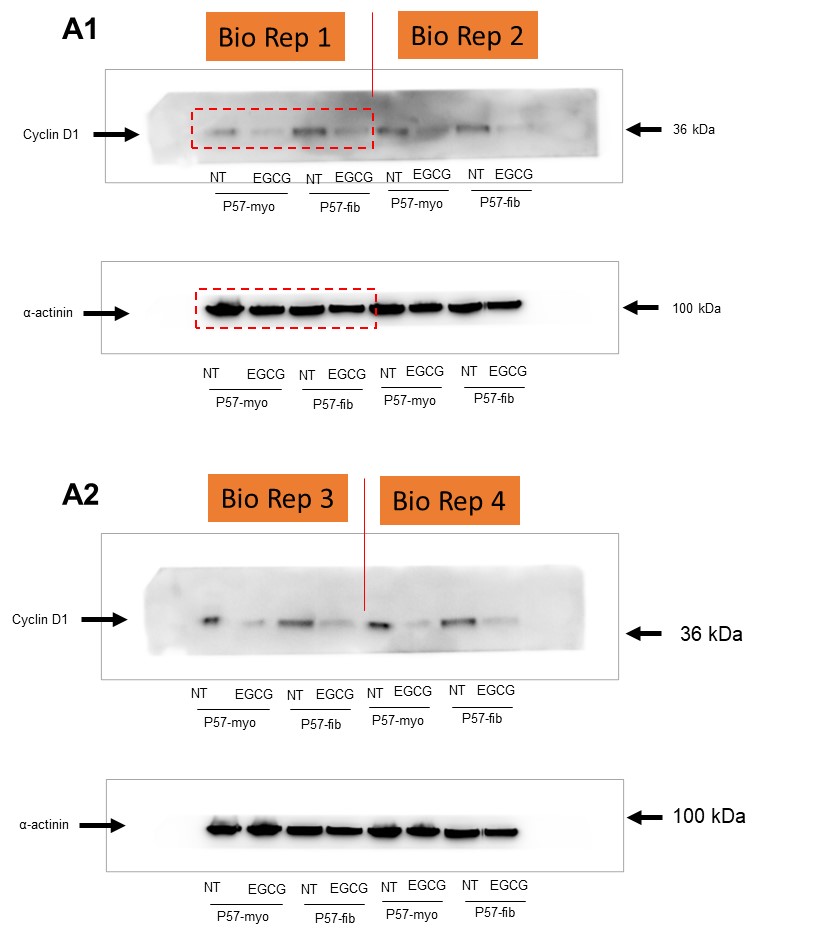


Figure S2. Full immunoblots related to Figure 1. Cyclin D1 and α-actinin in P57 myometrial and fibroid cells (A1-A2). Membranes were cut into several pieces (based on the molecular weight of proteins of interest) prior to hybridization with primary antibodies during blotting. NT=untreated control cells. Biological replicates (Bio Rep) of immunoblots are presented here used for analysis. MW (in kDa) are indicated. α-actinin was used as loading control to normalize the data.


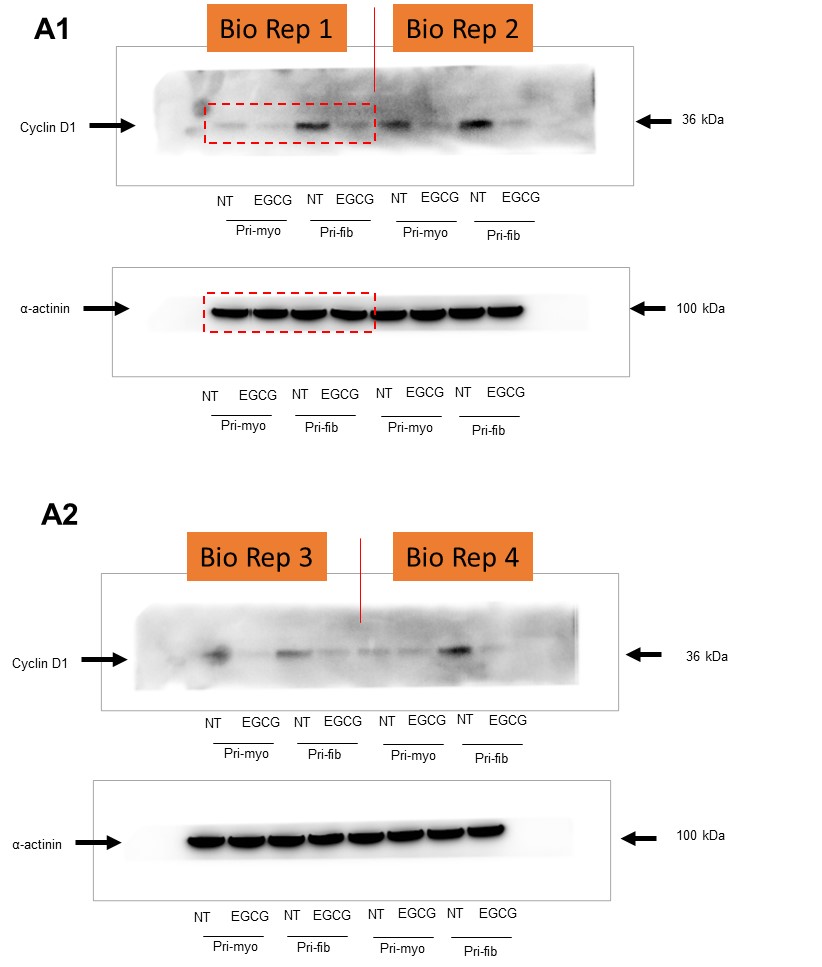


Figure S3. Full immunoblots related to Figure 1. Cyclin D1 and α-actinin in primary myometrial and fibroid cells (A1-A2). Membranes were cut into several pieces (based on the molecular weight of proteins of interest) prior to hybridization with primary antibodies during blotting. NT=untreated control cells. Biological replicates (Bio Rep) of immunoblots are presented here used for analysis. MW (in kDa) are indicated. α-actinin was used as loading control to normalize the data.


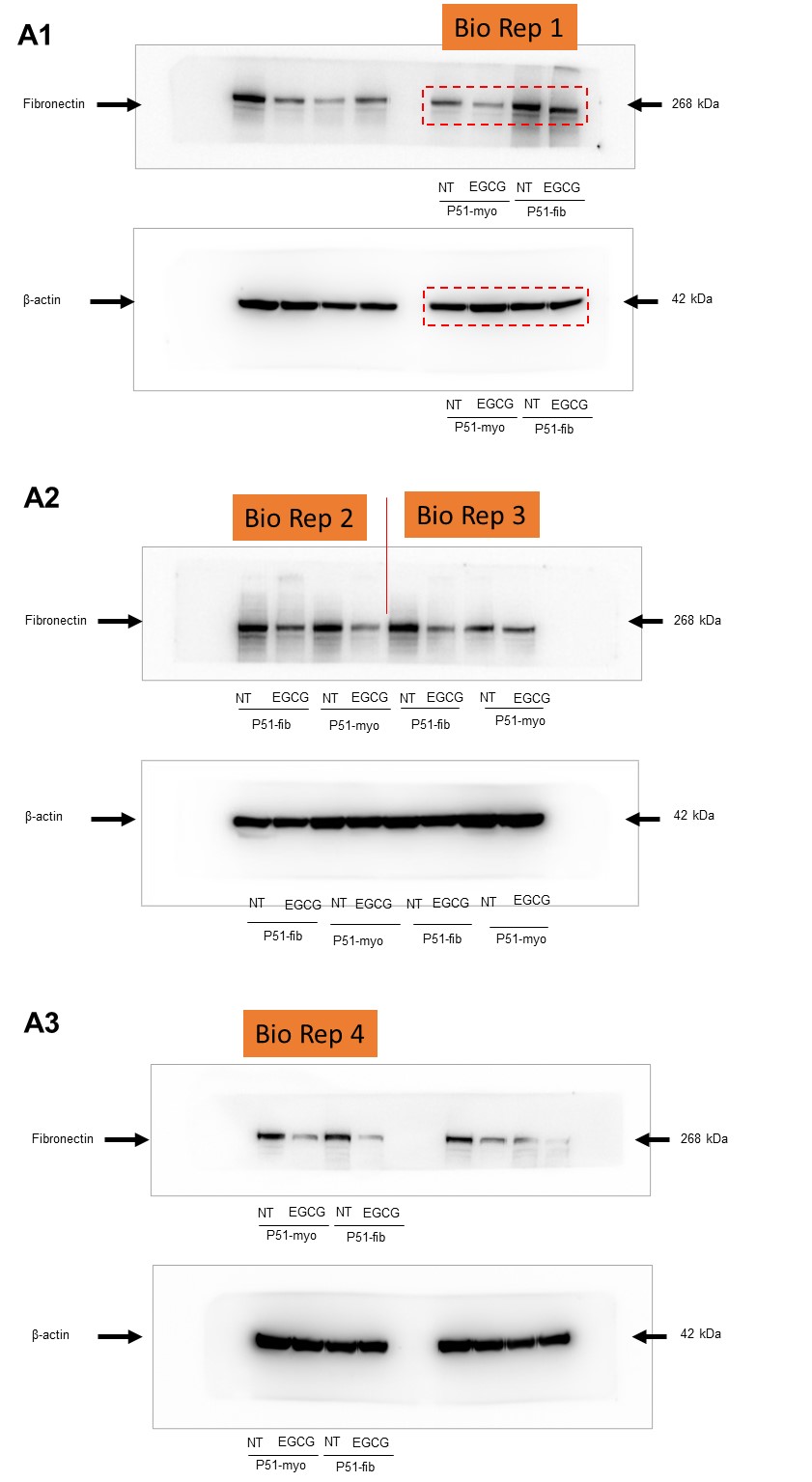


Figure S4. Full immunoblots related to Figure 2. Fibronectin and β-actin in P51 myometrial and fibroid cells (A1-A3). Membranes were cut into several pieces (based on the molecular weight of proteins of interest) prior to hybridization with primary antibodies during blotting. NT=untreated control cells. Biological replicates (Bio Rep) of immunoblots are presented here used for analysis. MW (in kDa) are indicated. β-actin was used as loading control to normalize the data.


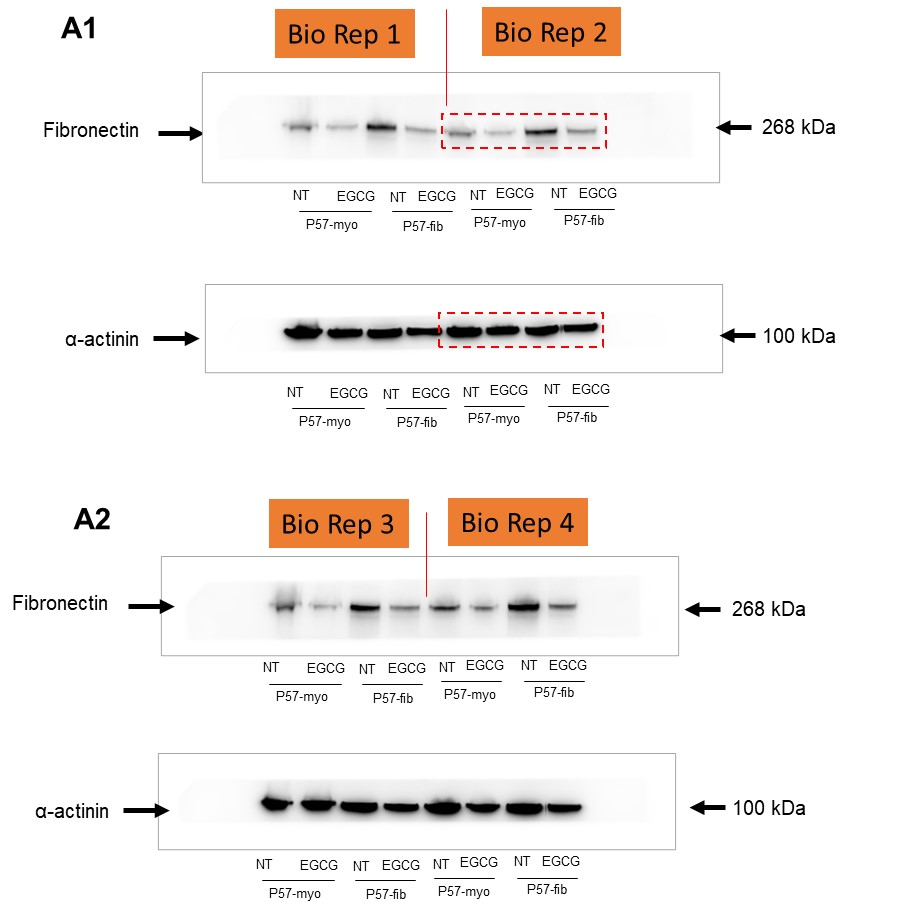


Figure S5. Full immunoblots related to Figure 2. Fibronectin and α-actinin in P57 myometrial and fibroid cells (A1-A2). Membranes were cut into several pieces (based on the molecular weight of proteins of interest) prior to hybridization with primary antibodies during blotting. NT=untreated control cells. Biological replicates (Bio Rep) of immunoblots are presented here used for analysis. MW (in kDa) are indicated. α-actinin were used as loading control to normalize the data.


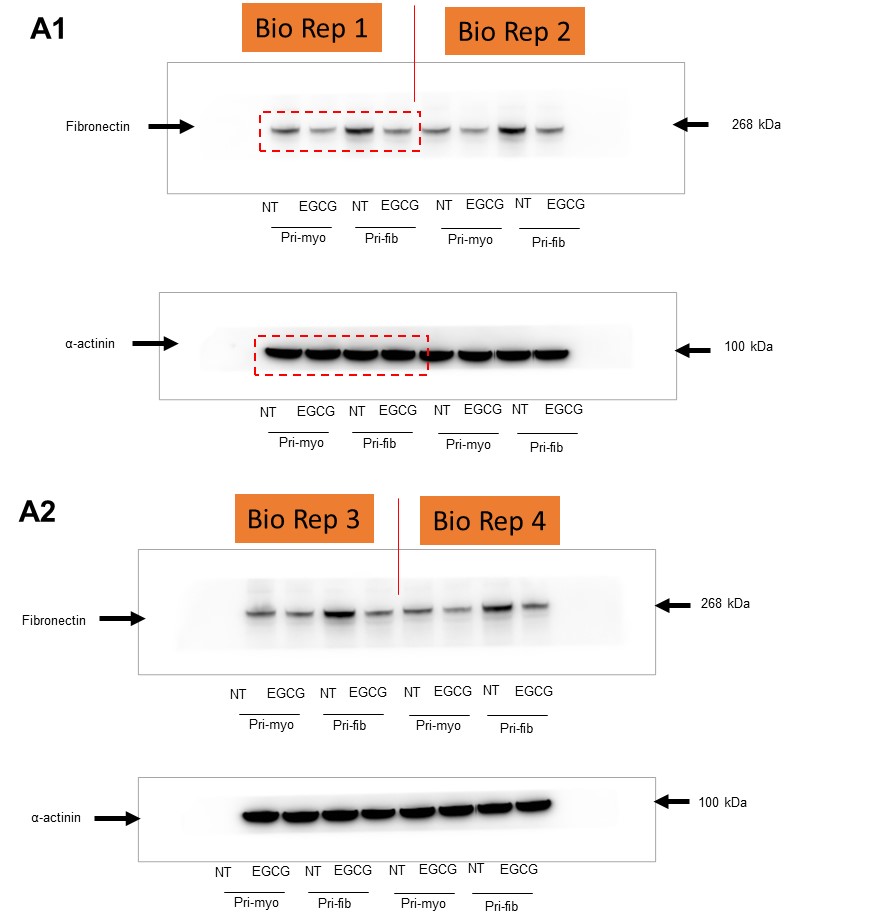


Figure S6. Full immunoblots related to Figure 2. Fibronectin and α-actinin in primary myometrial and fibroid cells (A1-A2). Membranes were cut into several pieces (based on the molecular weight of proteins of interest) prior to hybridization with primary antibodies during blotting. NT=untreated control cells. Biological replicates (Bio Rep) of immunoblots are presented here used for analysis. MW (in kDa) are indicated. α-actinin was used as loading control to normalize the data.


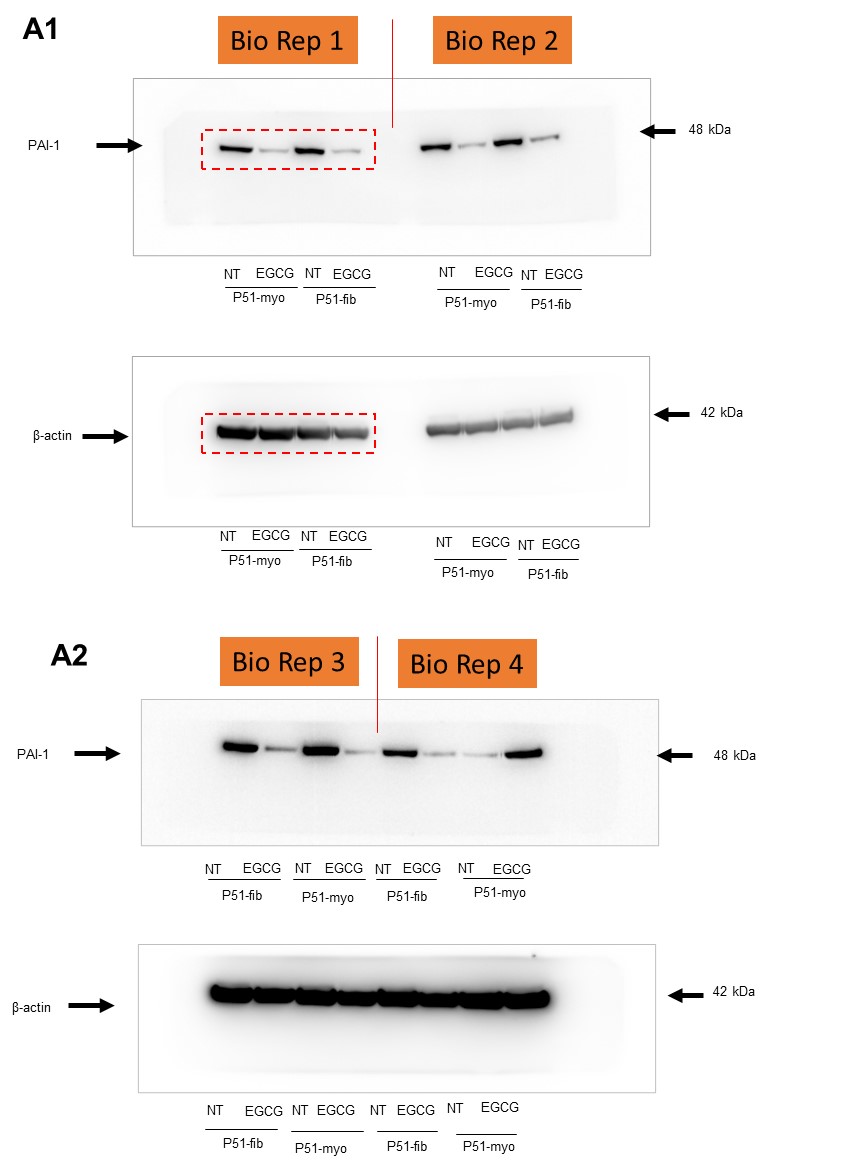


Figure S7. Full immunoblots related to Figure 3. PAI-1 and β-actin in P51 myometrial and fibroid cells (A1-A2). Membranes were cut into several pieces (based on the molecular weight of proteins of interest) prior to hybridization with primary antibodies during blotting. NT=untreated control cells. Biological replicates (Bio Rep) of immunoblots are presented here used for analysis. MW (in kDa) are indicated. β-actin was used as loading control to normalize the data.


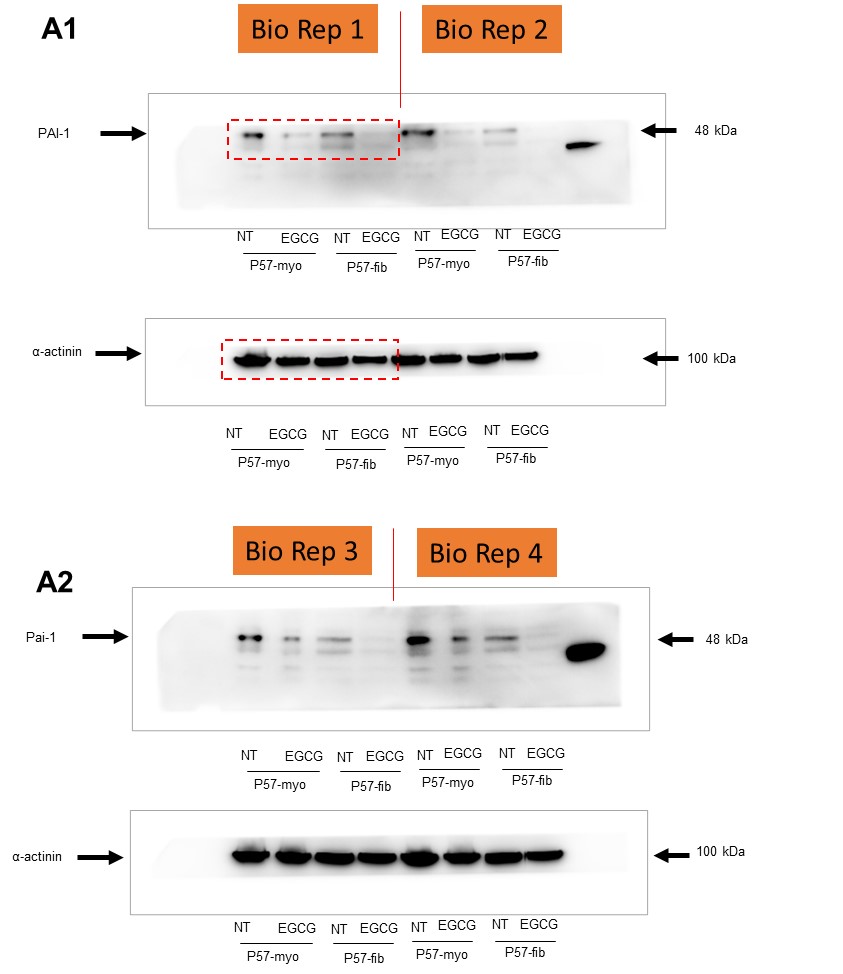


Figure S8. Full immunoblots related to Figure 3. PAI-1 and α-actinin in P57 myometrial and fibroid cells (A1-A2). Membranes were cut into several pieces (based on the molecular weight of proteins of interest) prior to hybridization with primary antibodies during blotting. NT=untreated control cells. Biological replicates (Bio Rep) of immunoblots are presented here used for analysis. MW (in kDa) are indicated. α-actinin were used as loading control to normalize the data.


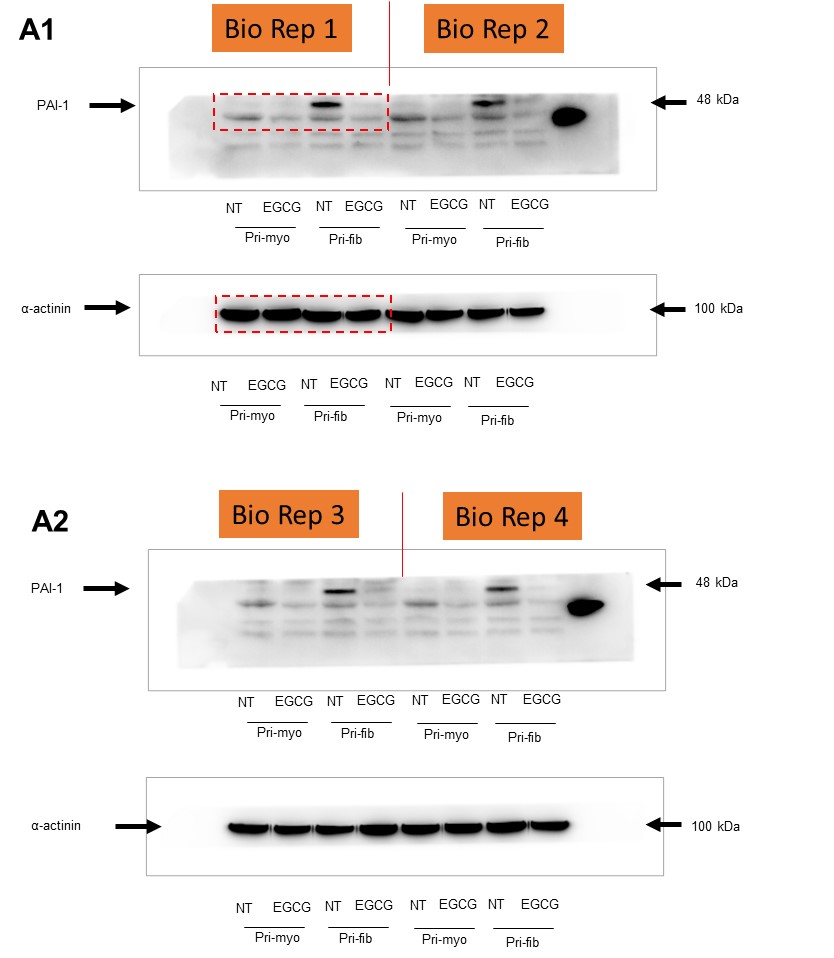


Figure S9. Full immunoblots related to Figure 3. PAI-1 and α-actinin in primary myometrial and fibroid cells (A1-A2). Membranes were cut into several pieces (based on the molecular weight of proteins of interest) prior to hybridization with primary antibodies during blotting. NT=untreated control cells. Biological replicates (Bio Rep) of immunoblots are presented here used for analysis. MW (in kDa) are indicated. α-actinin were used as loading control to normalize the data.


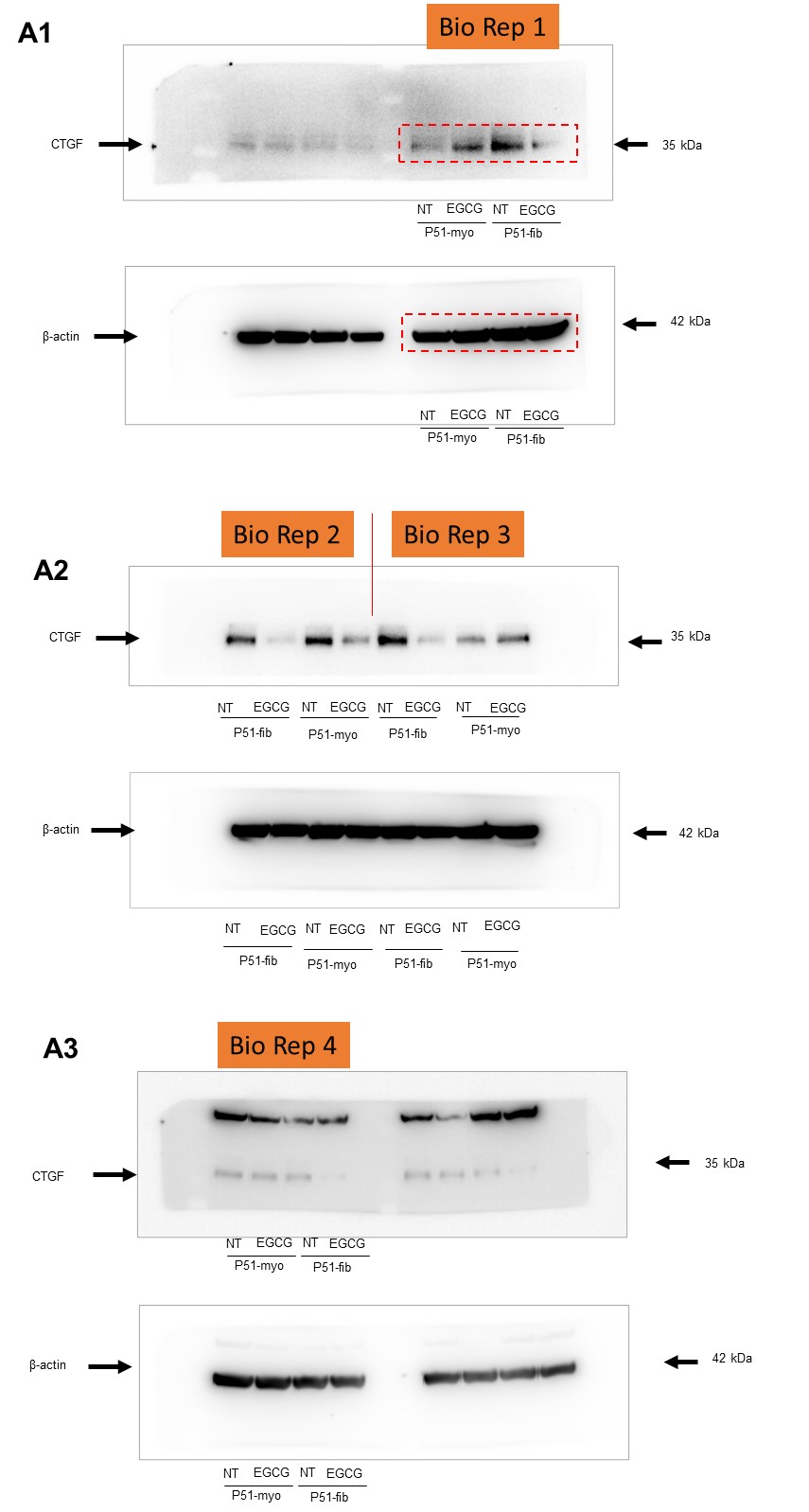


Figure S10. Full immunoblots related to Figure 4. CTGF and β-actin in P51 myometrial and fibroid cells (A1-A3). Membranes were cut into several pieces (based on the molecular weight of proteins of interest) prior to hybridization with primary antibodies during blotting. NT=untreated control cells. Biological replicates (Bio Rep) of immunoblots are presented here used for analysis. MW (in kDa) are indicated. β-actin was used as loading control to normalize the data.


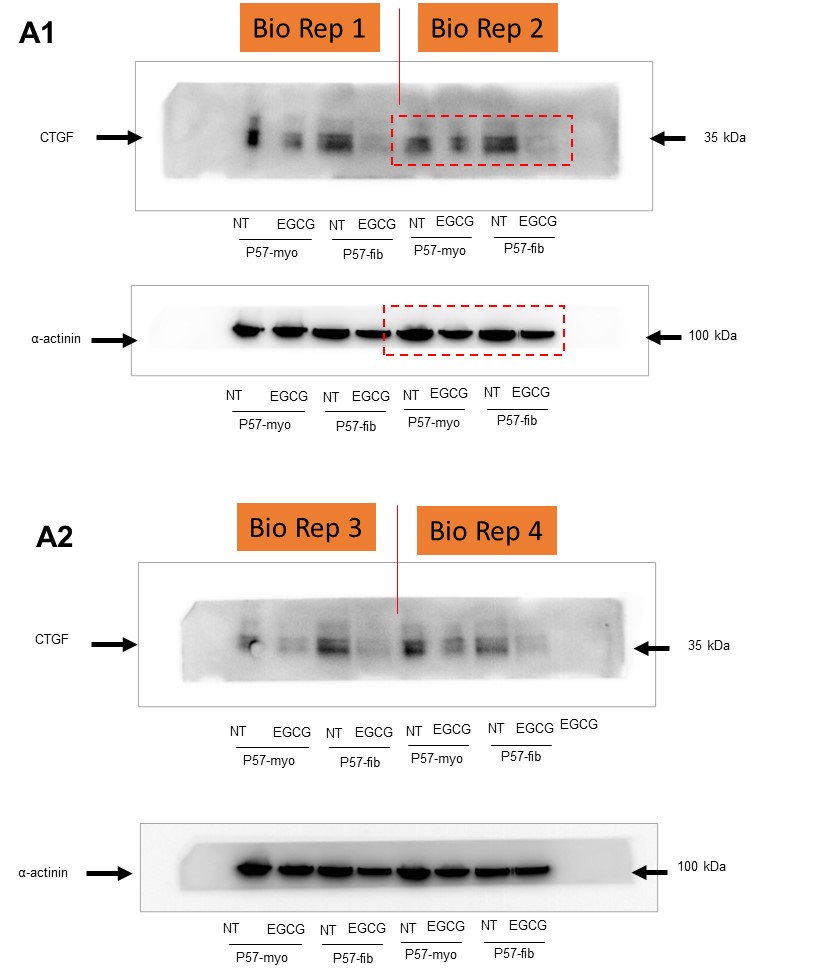


Figure S11. Full immunoblots related to Figure 4. CTGF and α-actinin in P57 myometrial and fibroid cells (A1-A2). Membranes were cut into several pieces (based on the molecular weight of proteins of interest) prior to hybridization with primary antibodies during blotting. NT=untreated control cells. Biological replicates (Bio Rep) of immunoblots are presented here used for analysis. MW (in kDa) are indicated. α-actinin was used as loading control to normalize the data.


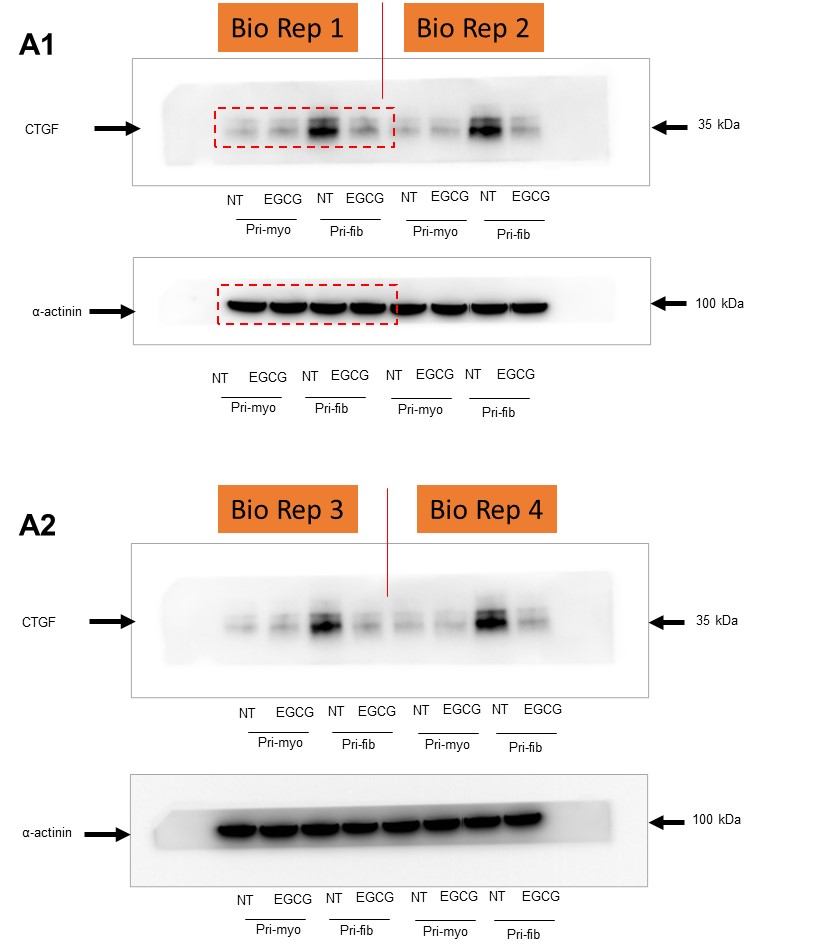


Figure S12. Full immunoblots related to Figure 4. CTGF and α-actinin in primary myometrial and fibroid cells (A1-A2). Membranes were cut into several pieces (based on the molecular weight of proteins of interest) prior to hybridization with primary antibodies during blotting. NT=untreated control cells. Biological replicates (Bio Rep) of immunoblots are presented here used for analysis. MW (in kDa) are indicated. α-actinin was used as loading control to normalize the data.


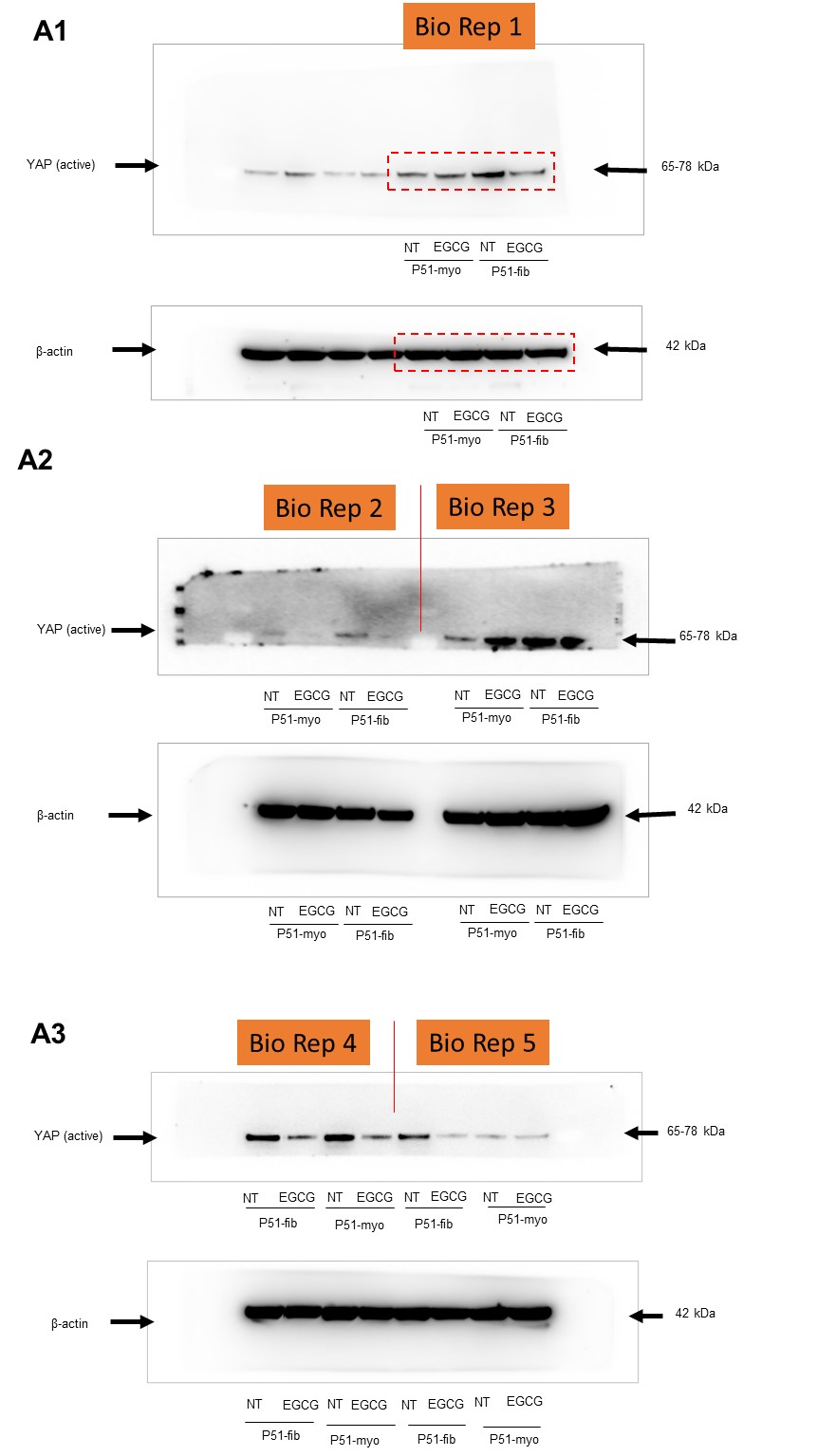


Figure S13. Full immunoblots related to Figure 5. YAP (active) and β-actin in P51 myometrial and fibroid cells (A1-A3). Membranes were cut into several pieces (based on the molecular weight of proteins of interest) prior to hybridization with primary antibodies during blotting. NT=untreated control cells. Biological replicates (Bio Rep) of immunoblots are presented here used for analysis. MW (in kDa) are indicated. β-actin was used as loading control to normalize the data.


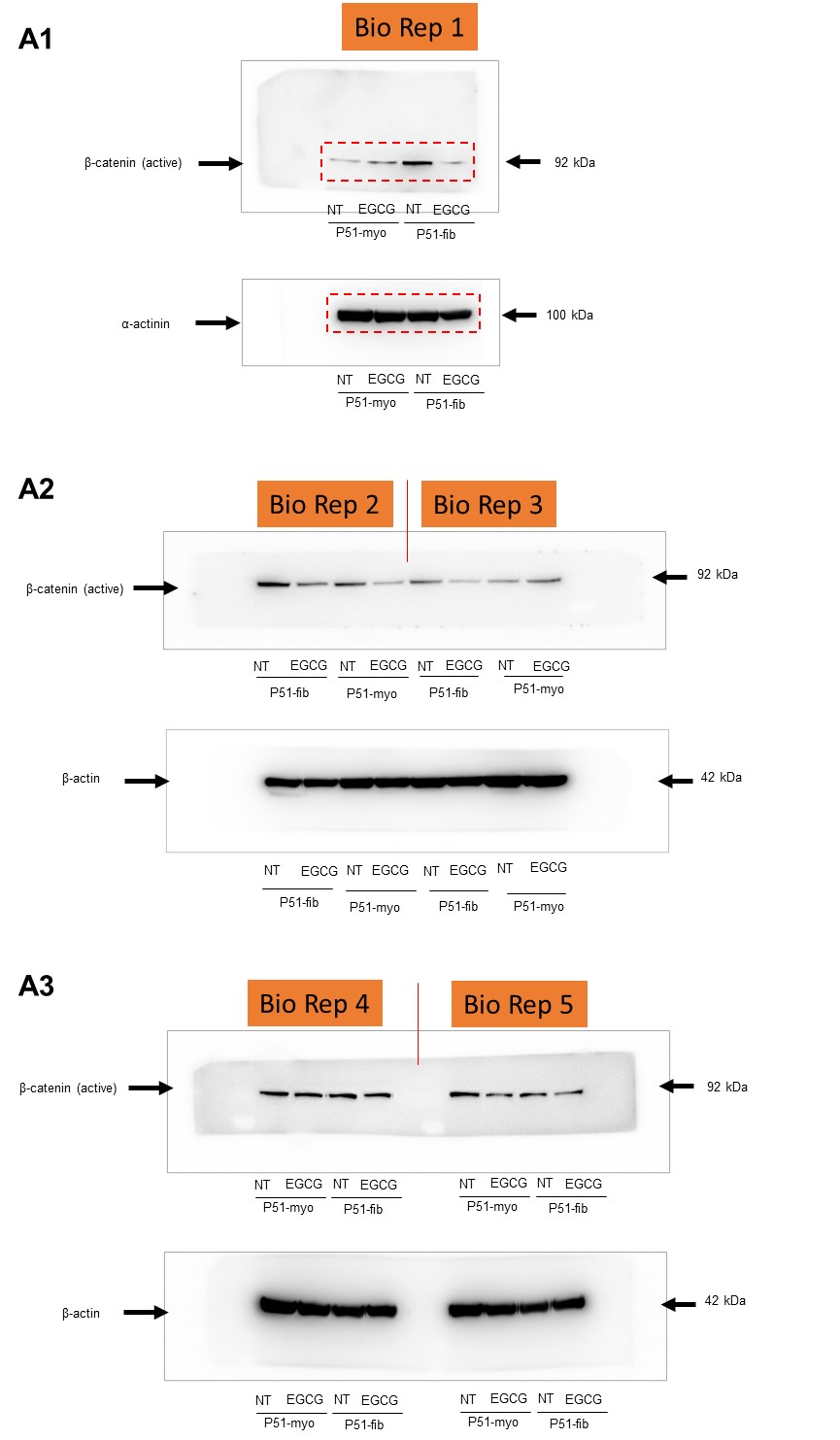


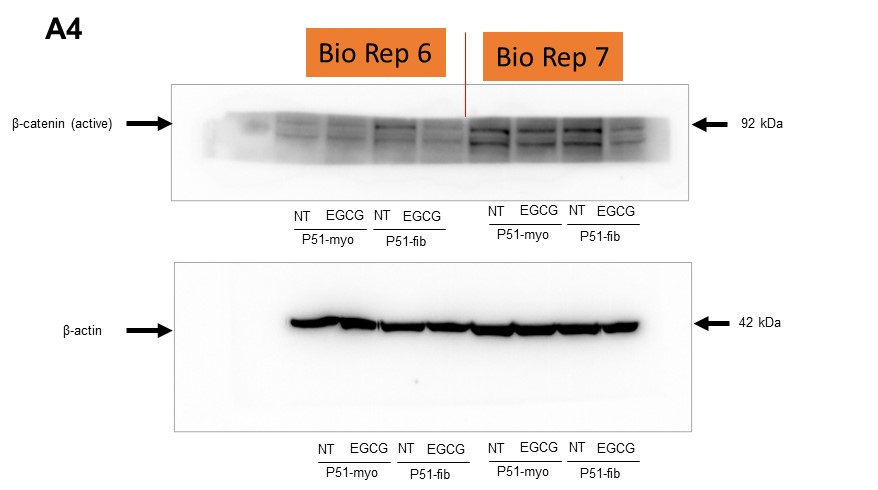


Figure S14. Full immunoblots related to Figure 5. β-catenin and β-actin in P51 myometrial and fibroid cells (A1-A4). Membranes were cut into several pieces (based on the molecular weight of proteins of interest) prior to hybridization with primary antibodies during blotting. NT=untreated control cells. Biological replicates (Bio Rep) of immunoblots are presented here used for analysis. MW (in kDa) are indicated. β-actin was used as loading control to normalize the data.


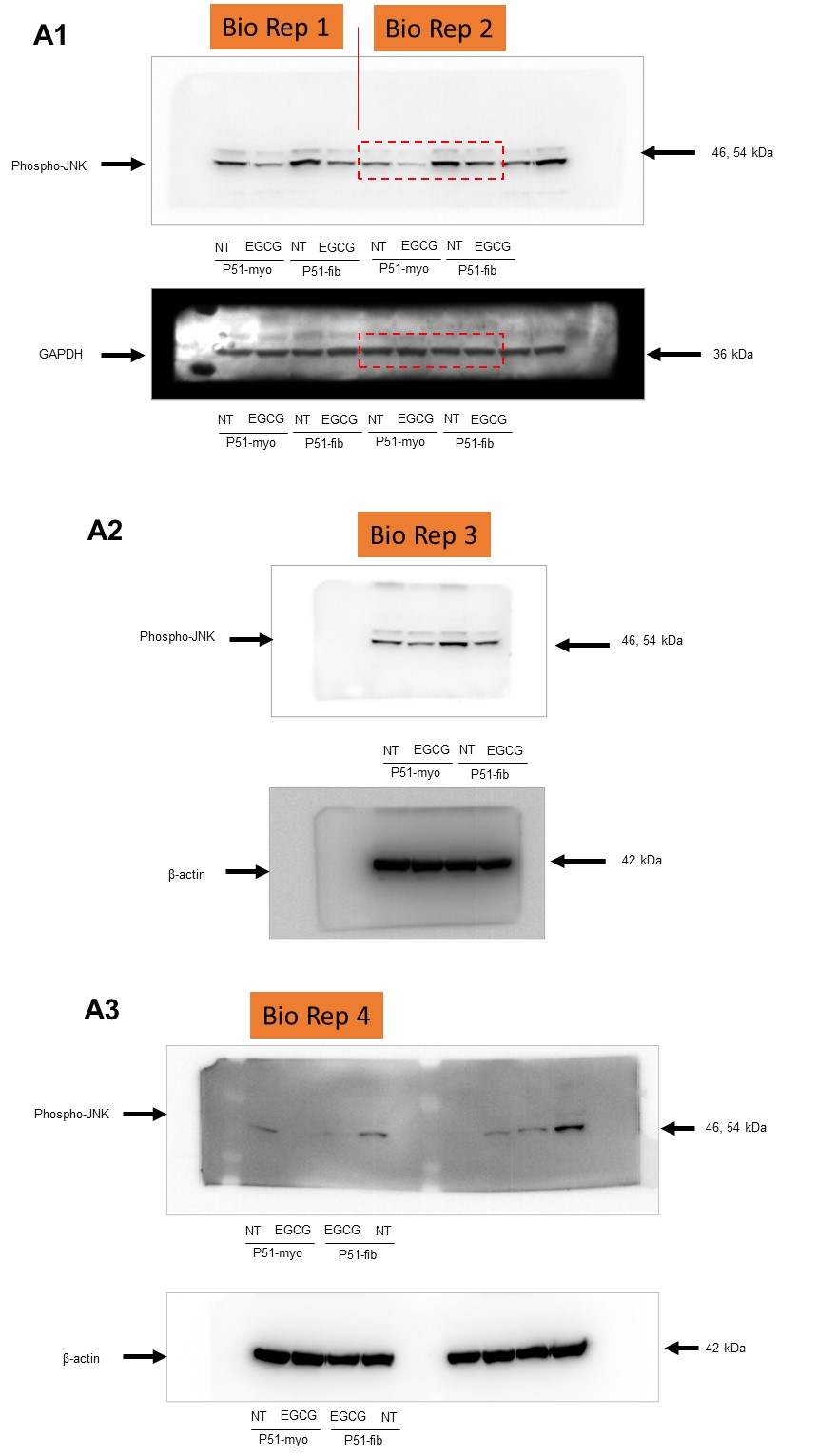


Figure S15. Full immunoblots related to Figure 5. Phospho-JNK and GAPDH in P51 myometrial and fibroid cells (A1-A3). Membranes were cut into several pieces (based on the molecular weight of proteins of interest) prior to hybridization with primary antibodies during blotting. NT=untreated control cells. Biological replicates (Bio Rep) of immunoblots are presented here used for analysis. MW (in kDa) are indicated. β-actin and GAPDH were used as loading controls to normalize the data.


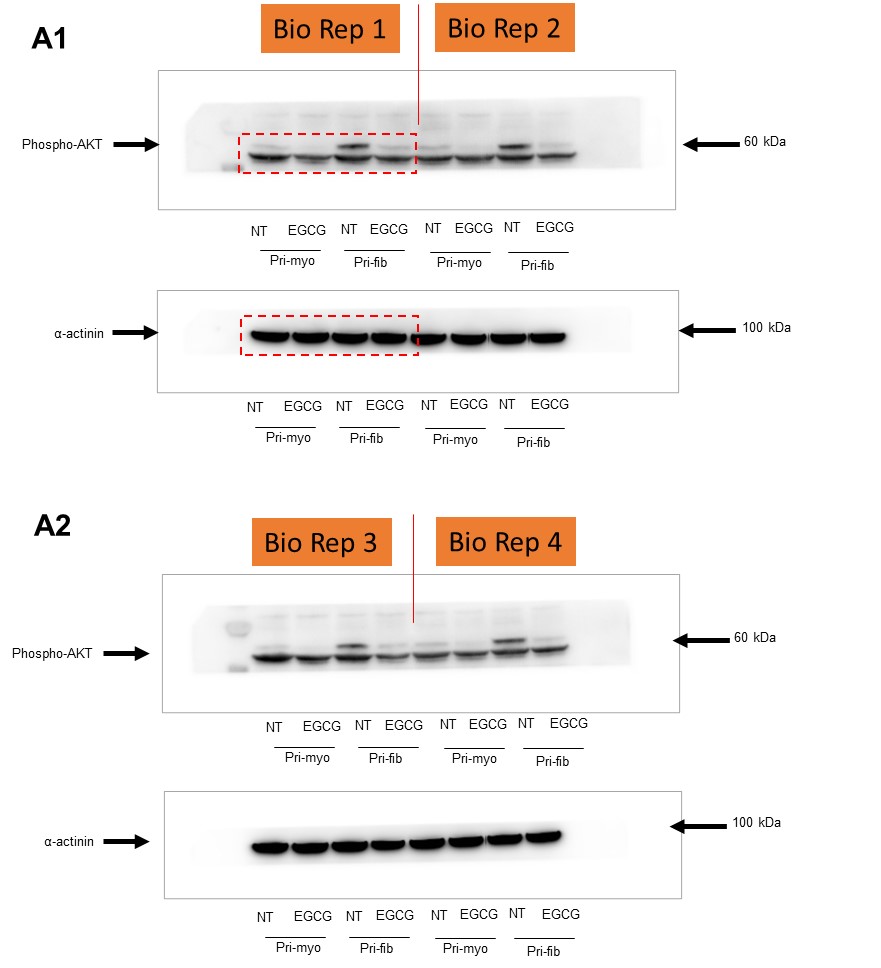


Figure S16. Full immunoblots related to Figure 5. Phospho-AKT and α-actinin in primary myometrial and fibroid cells (A1-A2). Membranes were cut into several pieces (based on the molecular weight of proteins of interest) prior to hybridization with primary antibodies during blotting. NT=untreated control cells. Biological replicates (Bio Rep) of immunoblots are presented here used for analysis. MW (in kDa) are indicated. α-actinin was used as loading control to normalize the data.


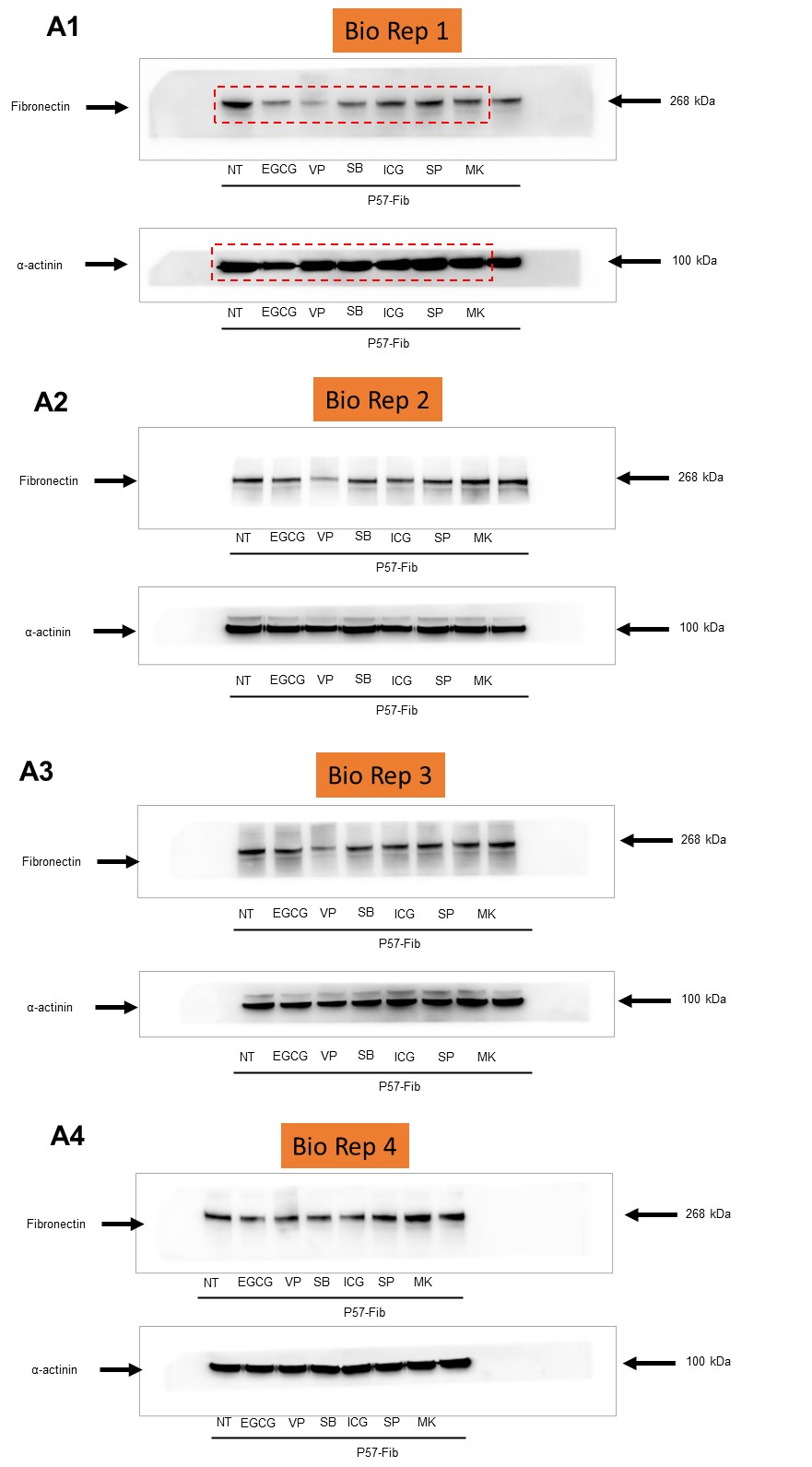


Figure S17. Full immunoblots related to Figure 6. Fibronectin and α-actinin in P57 fibroid cells (A1-A4). Membranes were cut into several pieces (based on the molecular weight of proteins of interest) prior to hybridization with primary antibodies during blotting. NT=untreated control cells. Biological replicates (Bio Rep) of immunoblots are presented here used for analysis. MW (in kDa) are indicated. α-actinin was used as loading control to normalize the data.


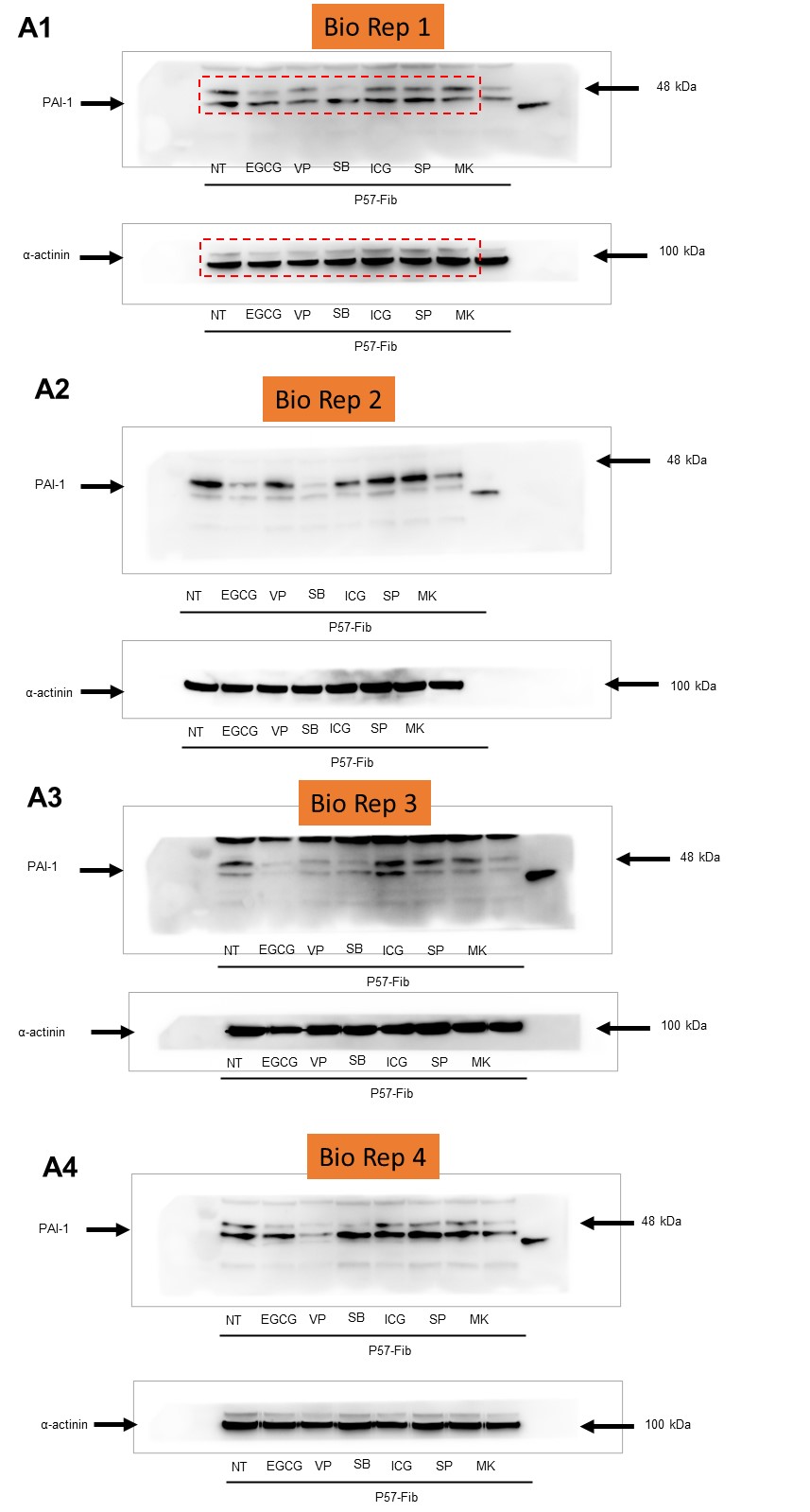


Figure S18. Full immunoblots related to Figure 6. PAI-1 and α-actinin in P57 fibroid cells (A1-A4). Membranes were cut into several pieces (based on the molecular weight of proteins of interest) prior to hybridization with primary antibodies during blotting. NT=untreated control cells. Biological replicates (Bio Rep) of immunoblots are presented here used for analysis. MW (in kDa) are indicated. α-actinin was used as loading control to normalize the data.


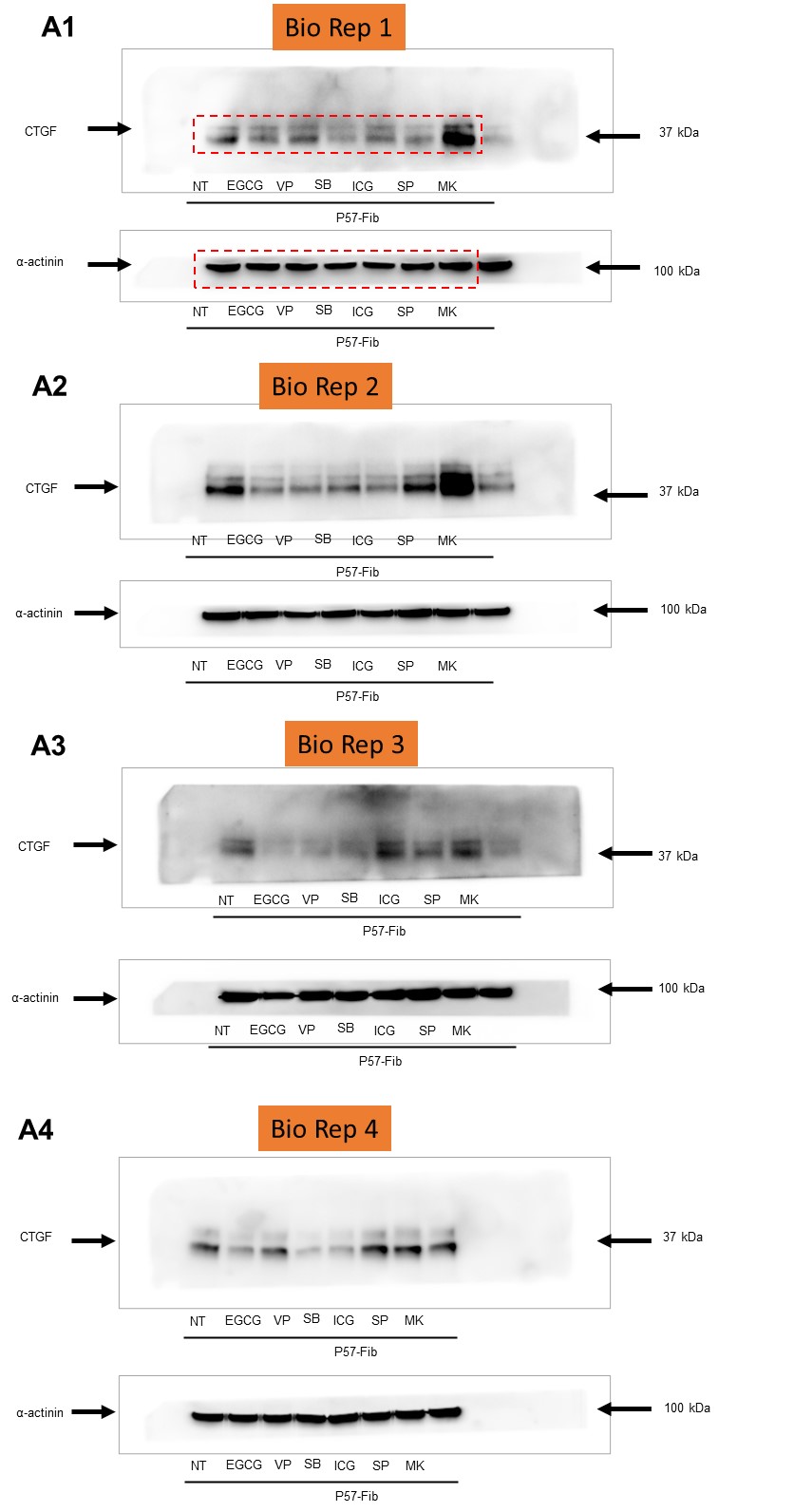


Figure S19. Full immunoblots related to Figure 6. CTGF and α-actinin in P57 fibroid cells (A1-A4). Membranes were cut into several pieces (based on the molecular weight of proteins of interest) prior to hybridization with primary antibodies during blotting. NT=untreated control cells. Biological replicates (Bio Rep) of immunoblots are presented here used for analysis. MW (in kDa) are indicated. α-actinin was used as loading control to normalize the data.


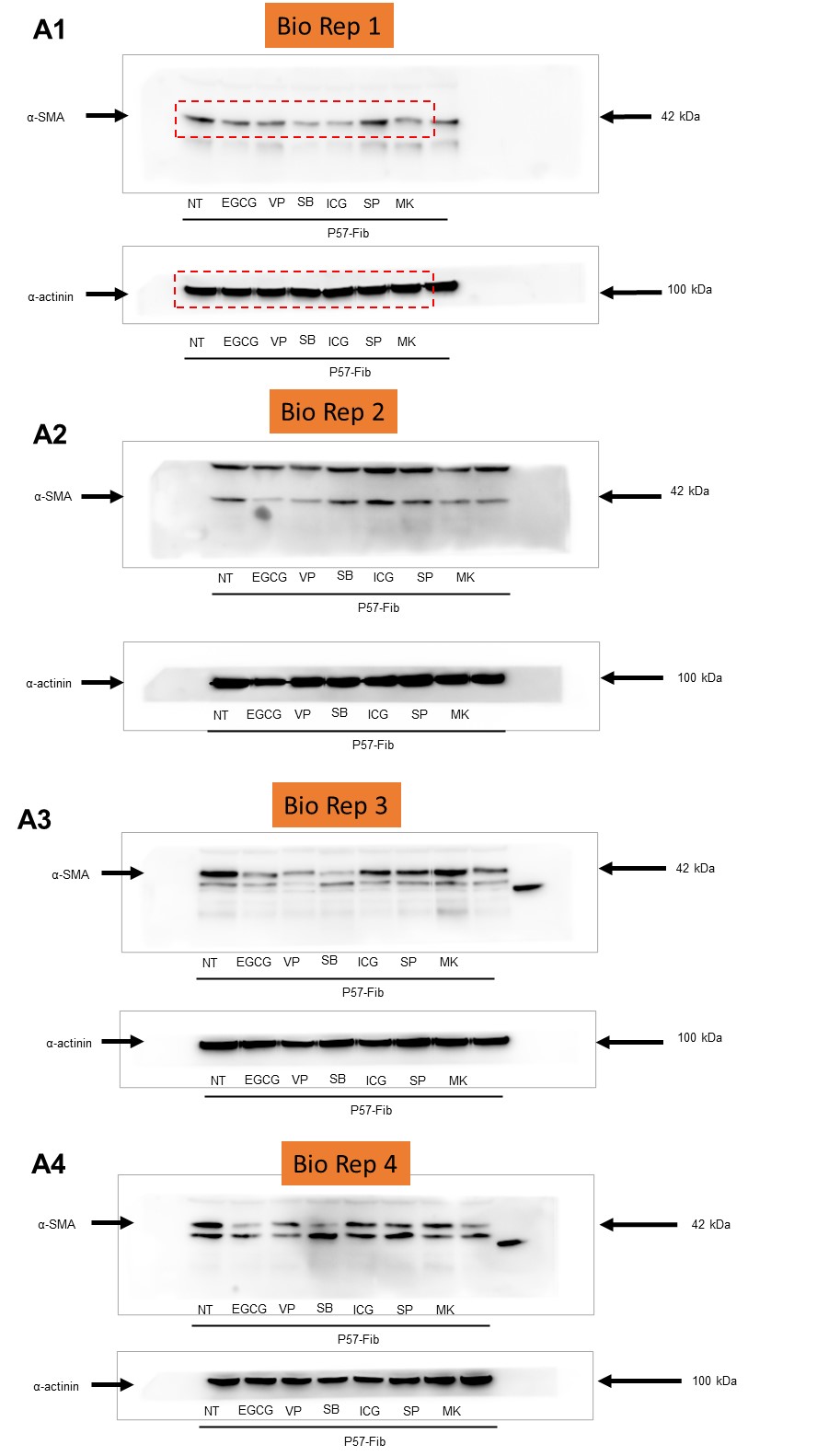


Figure S20. Full immunoblots related to Figure 6. α-SMA and α-actinin in P57 fibroid cells (A1-A4). Membranes were cut into several pieces (based on the molecular weight of proteins of interest) prior to hybridization with primary antibodies during blotting. NT=untreated control cells. Biological replicates (Bio Rep) of immunoblots are presented here used for analysis. MW (in kDa) are indicated. α-actinin was used as loading control to normalize the data.


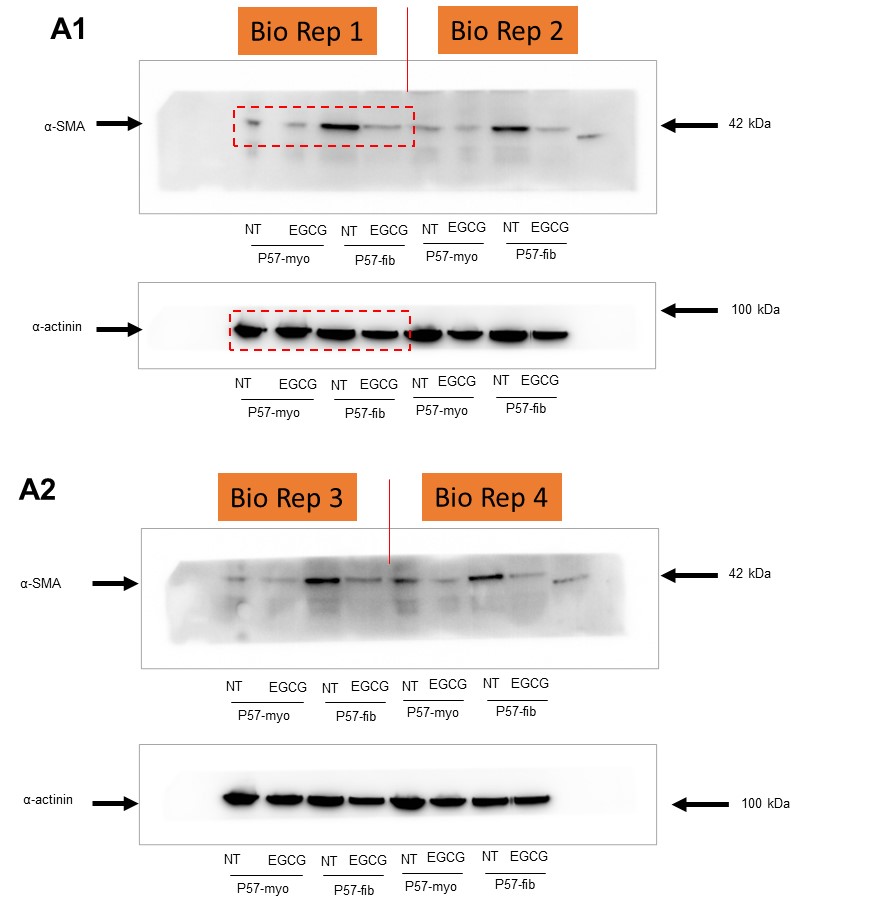


Figure S21. Full immunoblots related to Supplementary Figure 2. α-SMA and α-actinin in P57 myometrial and fibroid cells (A1-A2). Membranes were cut into several pieces (based on the molecular weight of proteins of interest) prior to hybridization with primary antibodies during blotting. NT=untreated control cells. Biological replicates (Bio Rep) of immunoblots are presented here used for analysis. MW (in kDa) are indicated. α-actinin was used as loading control to normalize the data.


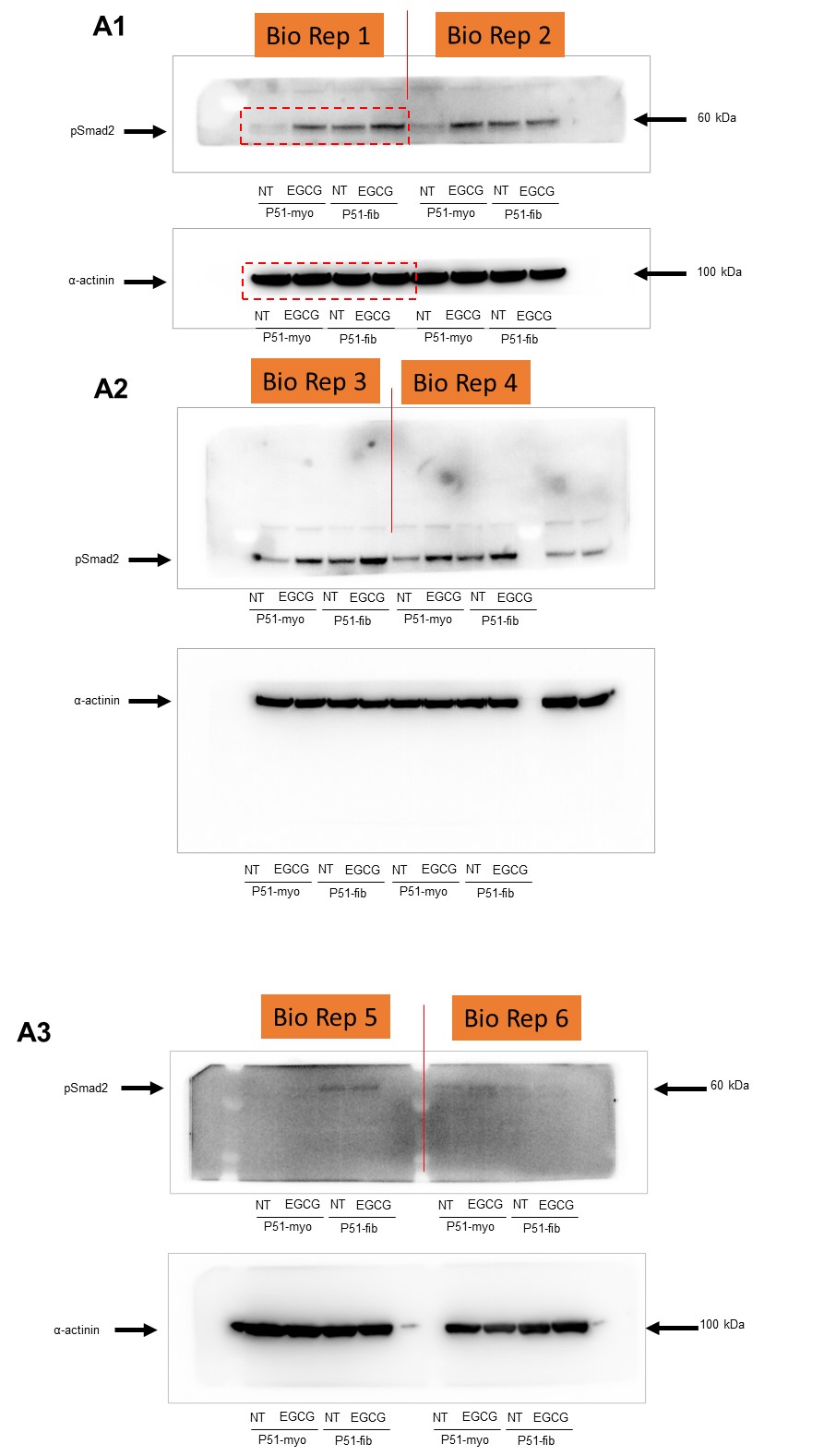


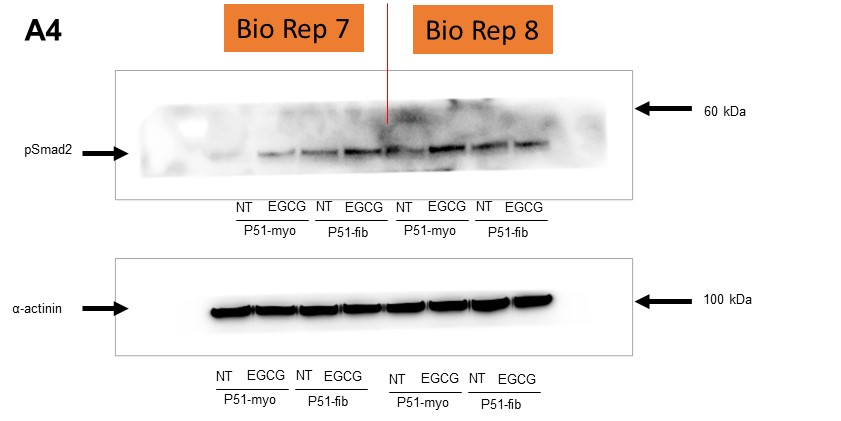


Figure S22. Full immunoblots related to Supplementary Figure 3. Phospho-Smad2 (Ser465/467) or pSmad2 and α-actinin in P51 myometrial and fibroid cells (A1-A4). Membranes were cut into several pieces (based on the molecular weight of proteins of interest) prior to hybridization with primary antibodies during blotting. NT=untreated control cells. Biological replicates (Bio Rep) of immunoblots are presented here used for analysis. MW (in kDa) are indicated. α-actinin was used as loading control to normalize the data.
